# Influence of the Reference Electrode on the Performance of Single‐Electrode Triboelectric Nanogenerators and the Optimization Strategies

**DOI:** 10.1002/advs.202206950

**Published:** 2023-04-23

**Authors:** Zetong Chen, Keren Dai, Jiaxiang Chen, Jingting Zhuo, Danna Zhao, Rui Ma, Xujing Zhang, Xubiao Li, Xiaofeng Wang, Guowei Yang, Fang Yi

**Affiliations:** ^1^ School of Materials Science and Engineering Nanotechnology Research Center Guangzhou Key Laboratory of Flexible Electronic Materials and Wearable Devices, State Key Laboratory of Optoelectronic Materials and Technologies Sun Yat‐sen University Guangzhou 510275 P. R. China; ^2^ School of Mechanical Engineering Nanjing University of Science and Technology Nanjing 210094 P. R. China; ^3^ Department of Precision Instrument Beijing Advanced Innovation Center for Integrated Circuits Tsinghua University Beijing 100084 P. R. China

**Keywords:** reference electrode, structure optimization, triboelectric nanogenerator

## Abstract

Owing to their unique advantages, single‐electrode triboelectric nanogenerators (SETENGs) have gained wide attention and have been applied in myriad areas, especially in the burgeoning flexible/wearable electronics. However, there is still a lack of a clear understanding of SETENGs. For example, previous simulation models generally put the reference electrode perpendicularly below the working part, but in practice, the reference electrode is designed in various scenarios and noticeable differences in outputs often occur when the reference electrode changes. With SETENGs developing towards wearability and portability, its reference electrode is often required to be constructed inside the device. Consequently, to achieve optimum performance, it is essential to understand the reference electrode's influence on the outputs. Here, the influence of the reference electrode on the performance of SETENGs is systematically investigated and the targeted optimization strategies are thoroughly revealed. First, theoretical simulations are conducted to investigate the reference electrode's effect on the performance of SETENGs with different structures and in various working modes. Secondly, the theoretical results are certified through corresponding experiments. Based on the results, the targeted optimization strategies for SETENGs are comprehensively demonstrated. This work provides fundamental guidance for the development of TENGs and the design and fabrication of new electronic devices.

## Graphical abstract

The influence of the reference electrode on the performance of single‐electrode triboelectric nanogenerators is systematically investigated and the targeted optimization strategies are thoroughly revealed.

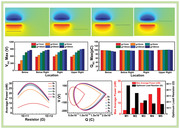

## 1 Introduction

With the rapid growth of energy consumption and the tightening supplies of energy, harvesting energy from the ambient environment has been given more and more attention around the world.^[^ ^1^, ^2^, ^3^, ^4^ ^]^ Mechanical energy, as a ubiquitous form of energy in nature, has shown tantalizing prospects due to its characteristics of cleanness and universal availability. Compared to other mechanical energy harvesting techniques,^[^ ^5^, ^6^, ^7^ ^]^ triboelectric nanogenerators (TENGs) have been experiencing explosive development owing to their merits of high efficiency, convenient fabrication, low cost, wide selection of materials, and so on.^[^ ^8^, ^9^, ^10^, ^11^ ^]^ The working mechanism of TENGs is based on the coupling of contact electrication and electrostatic induction.^[^ ^12^, ^13^, ^14^, ^15^, ^16^ ^]^ Depending on the mechanism of charge separation, TENGs can be divided into two basic modes: contact/separation mode based on vertical charge polarization^[^ ^17^, ^18^, ^19^, ^20^ ^]^ and sliding mode based on in‐plane charge polarization.^[^ ^21^, ^22^, ^23^ ^]^ Specifically, four types of TENGs have been successively proposed and demonstrated, namely the vertical‐contact/separation TENGs, lateral‐sliding TENGs, single‐electrode TENGs (SETENGs) and freestanding‐triboelectric‐layer TENGs (FTENGs).^[^ ^24^, ^25^, ^26^, ^27^, ^28^, ^29^, ^30^ ^]^ Up to now, substantial efforts have been made to promote the output performance and applications of TENGs. Notably, by providing comprehensive optimization design of device structures, materials, and load circuits, modeling and simulations have made great contributions to the improvement of the output performance of TENGs.^[^ ^31^, ^32^, ^33^, ^34^, ^35^, ^36^, ^37^, ^38^ ^]^

Among the different types of TENGs, SETENGs have attracted intensitve attention due to their advantages including easy fabrication, simple structure, and one freely moving component, which render them favorable for various applications, especially in the burgeoning field of flexible/wearable electronics. However, there is still a lack of a clear understanding of the working mechanisms of SETENGs. Specifically, in practice, the reference electrode is usually not fixed (in location, size, or shape) and noticeable differences in the electrical outputs often occur when different reference electrodes are connected. Furthermore, previous modeling and optimization studies of SETENGs have adopted an idealized assumption that the reference electrode is located perpendicularly below the primary electrode (i.e, the working electrode) with the same size^[^ ^33^, ^34^, ^35^ ^]^; but such an idealized assumption hardly matches actual situations. With the development of SETENGs towards portability and wearability, the reference electrode generally needs to be fabricated inside the device together with one fixed working part,^[^ ^39^, ^40^, ^41^ ^]^ in which case, the influence of the reference electrode on the performance cannot be ignored. In addition, although it is often found that there occurs variations in the electrical outputs in experiments or practical demonstrations when the connected reference electrode is different, the correlated mechanisms have not yet been thoroughly revealed. Therefore, there is a strong and urgent need to fully understand the effect of the reference electrode on the output performance of SETENGs, which is of crucial importance for the comprehensive optimization design of practical devices.

In this work, theoretical models are constructed to systematically study the effect of the reference electrode on output performance of SETENGs, and the corresponding experiments verify the theoretical simulation results. Based on the obtained results, the working mechanisms are exhaustively analyzed through numerical calculation and derivation methods. Moreover, the targeted optimization strategies for SETENGs are comprehensively revealed.

The rest of this paper is organized as follows. Section 2 focuses on the theoretical investigations. First, the contact/separation‐mode SETENGs structured with the primary electrode serving as the triboelectric layer (P‐CS‐SETENG) are studied, regarding the influences and optimization strategies of the reference electrode's location, size, and shape. Then, the influence analysis and optimal design of the reference electrode for sliding‐mode SETENGs structured with the primary electrode serving as the triboelectric layer (P‐S‐SETENG) are examined. Thirdly, the investigation is extended to SETENGs structured with a dielectric layer attached onto the primary electrode (D‐SETENGs), and a comparison with the results obtained from the P‐SETENG (P‐CS‐SETENG and P‐S‐SETENG) is analyzed. Section 3 presents the experimental verification of the above theoretical analysis results. Section 4 summarizes the results and discussions. Section 5 provides detailed information for the experiments.

## 2 Theoretical Investigation

### 2.1 P‐CS‐SETENG Working by Contact/Separation Motion

The physical model of the P‐CS‐SETENG was constructed on the simulation software COMSOL. The model consists of three parts: the contacting object, the primary electrode, and reference electrode of the P‐CS‐SETENG. The contacting object and primary electrode are stacked face to face to form the pair of triboelectric layers. The generated charges on the triboelectric layers after contact and separation process are uniformly distributed on the surface with negligible decay, in accord with the fact that surface triboelectric charges of dielectric polymers can maintain for a long time.^[^ ^42^, ^43^ ^]^ Owing to the coupling effect of contact electrification and electrostatic induction, the two electrodes produce potential differences that drive electrons to flow through the external load when there occurs relative motion between the two triboelectric layers. Therefore, the output voltage in the open circuit (*V*_OC_) and transferred charges in the short circuit (*Q*_SC_) with separation distances (*x*) can be calculated. More details of the theoretical simulation model for the P‐CS‐SETENG can be found in Note S1, Supporting Information.

#### 2.1.1 The Effect of the Reference Electrode Location

In previous research, the reference electrode is always placed directly below the primary electrode.^[^ ^33^, ^34^, ^35^ ^]^ However, this assumption is over‐idealized since the reference electrode is not fixed (in location, size or shape) in practice. In this section, the effect of the reference electrode's location, including the direction and gap distance (g) from the primary electrode, on the output performance of the P‐CS‐SETENG will be investigated.

**Figure 1:**
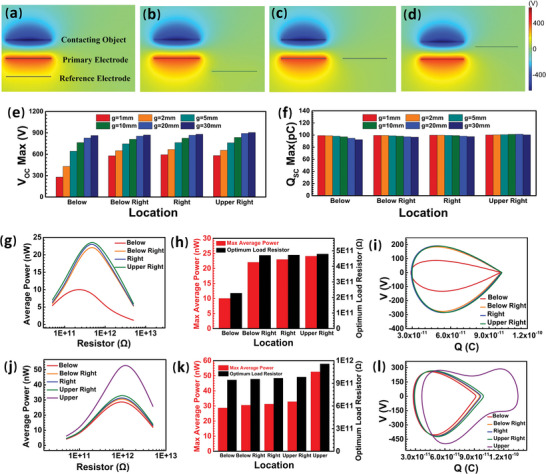
The effect of the reference electrode's location on the output performance of the P‐CS‐SETENG. a‐d) Images showing the reference electrode placed at (a) below, (b) below right, (c) right, and (d) upperright of the primary electrode. e‐f) The (e) V_OCmax_ and (f) *Q*_SCmax_ with the reference electrode at different locations under different gaps between the primary and reference electrodes. g‐i) The (g) average power, (h) maximum average power and optimum load resistor, and (i) *V–Q* plots under optimum load resistors when the reference electrode is at different locations (*g* = 1 mm). j‐l) The (j) average power, (k) maximum average power and optimum load resistor, and l) *V–Q* plots under optimum load resistors when the reference electrode at different locations (*g* = 30 mm).

As shown in **Figure** **1**a‐d, the reference electrode with different directions are labeled as “below”, “below right”, “right” and “upper right”, respectively. Due to the symmetrical structure of the device, the situation on the left side is the same as that on the right side, and the situation of placing the reference electrode directly above the primary electrode is similar to the contact/separation‐mode FTENG^[^ ^31^ ^]^; so neither the former situation nor the latter situation when the separation displacement (*x*) is larger than the gap distance are examined here. As shown in Figure 1e‐f, the *V*_OCmax_ and *Q*_SCmax_ are calculated with different gaps and different directions under a maximum separation displacement (*x*_max_) of 28 mm. The values of *V*_OCmax_ calculated at below right, right, and upper right are significantly higher than those at below (2.13, 2.10, and 2.07 times higher, respectively), but the differences in *Q*_SCmax_ calculated at different directions are relatively small, which are only 1%, 0.5% and 0.3% higher than those at below. These results indicate the possibility of lifting the output power of the P‐CS‐SETENG by placing the reference electrode with a horizontal bias.

The effect of the reference electrode's direction on the *V*_OC_ and *Q*_SC_ of the P‐CS‐SETENG can be interpreted utilizing the electric field and potential distribution at different locations. The surfaces of the contacting object and primary electrode carry charges of the same magnitude but opposite signs after contact. These two triboelectric layers are equivalent to a capacitor, and the strength of the electric field around it with the reference electrode at different locations reveals that the primary electrode possesses the highest potential (Figure S1, Supporting Information). Due to the inherent physical properties of the electric field of the parallel plate capacitor, the potential decreases with a relatively gentle trend below the primary electrode; in contrast, on the right or left side of it, the potential decreases with a relatively steep trend (Figure S1, Supporting Information). Such potential distribution indicates that when the reference electrode is located below right, right, and up right of the original electrode, its potential will be lower and there will be higher potential differences between the primary and reference electrodes than those located at directly below (Figure S1b‐e, Supporting Information). For the P‐CS‐SETENG, its open‐circuit voltage (*V*_OC_) equals the potential difference between the primary electrode (*φ*_1_) and reference electrode (*φ*_2_):

(1) $$V_{OC}=\varphi_{1}\;-\varphi_{2}$$

Therefore, the P‐CS‐SETENGs with a horizontally‐biased reference electrode have higher V_OC_ than that with a reference electrode directly below the primary electrode. In addition, due to the contribution of negative triboelectric charges on the contacting object above, upward movement of the horizontally‐biased reference electrode is beneficial for it to achieve a lower potential and thereby a larger potential difference from the primary electrode. The above analyses clarify why the V_OC_ calculated at different directions ranks from the largest to the smallest as follows: upper right, right, below right, and below.

It can also be found from Figure 1e‐f that with *g* increasing from 1 mm to 30 mm, the *V*_OCmax_ calculated at the below, below right, right, and upper right increase to 3.09, 1.51, 1.48 and 1.56 times of the original values, respectively. Note that the calculated *V*_OC_ and *Q*_SC_ with changing separation displacement *x* at various *g* are presented in Figure S2, Supporting Information This is also caused by the electric field and potential distribution (Figure S1, Supporting Information): moving down away from the primary electrode is beneficial for the reference electrode to have a lower potential, thus increasing the *V*_OC_. With the increasing g, the *Q*_SCmax_ is nearly unchanged when the reference electrode is located at the upper right, and slightly decreases when the reference electrode is located at the below, below right, and right (e.g., decreasing by 6.7%, 3%, and 2.4% at *g* = 30 mm compared with at *g* = 1 mm, respectively).

Furthermore, it is also found that the effect of the reference electrode's direction on the V_OC_ of the P‐CS‐SETENG wanes with the increase in *g*, while the effect on *Q*_SC_ hardly changes. When *g* = 1 mm, the *V*_OCmax_ with the reference electrode at upper right is 2.13 times that at below, and the values are 1.55, 1.21, 1.1, 1.08, and 1.05 times when at *g* = 2 mm, 5 mm, 10 mm, 20 mm, and 30 mm, respectively (Figure 1e). As for the *Q*_SCmax_, when *g* = 1 mm, the *Q*_SCmax_ with the reference electrode at upper right is 1% higher than that at below, and the values are 1.4%, 2.5%, 4.3%, 6.9%, and 8.5% at *g* = 2 mm, 5 mm, 10 mm, 20 mm, and 30 mm, respectively (Figure 1f). This changing trend can also be explained by the potential at different locations in the electric field. With *g* increases, the equipotential lines in the electric field far away from the primary electrode become sparser and more independent of locations, leading to smaller differences in *V*_OC_ (Figure S1, Supporting Information).

To sum up, the output performance of the P‐CS‐SETENG is greatly affected by the direction of the reference electrode when *g* is small, and the effect gradually decreases as *g* increases. Thus, to comprehensively examine the structure optimization strategy for SETENGs, two cases of small gap (*g* = 1 mm) and large gap (*g* = 30 mm) are thoroughly analyzed, respectively.

Set *g* = 1 mm, and the maximum voltage and maximum current under different load resistors with the reference electrode in different directions are calculated (Figure S3, Supporting Information). The maximum voltages ranking from the largest to the smallest are as follows: upper right, right, below right, and below (Figure S3a, Supporting Information), where the differences among them are significant under medium and high load resistances. The maximum currents sorted from the largest to the smallest are in the same order as the maximum voltages (Figure S3b, Supporting Information), where the differences among them are significant under low and medium load resistances, too. Consequently, the average powers from the largest to the smallest are also sorted as: upper right, right, below right, and below, where the differences among them are especially significant under the medium resistor loads (Figure S3c, Supporting Information). This is meaningful because the optimum load resistors matching the output maximum average powers are just in this medium range (Figure 1g). The maximum average powers with the reference electrode located at the upper right, right, and below right are 2.40, 2.30, and 2.20 times higher than that at the below, and the corresponding optimal load resistor resistances are 2.11, 2.08, and 2.07 times higher, respectively (Figure 1h). The corresponding output voltages (*V*) and transferred charges (*Q*) of the P‐CS‐SETENG with the optimum load resistors are shown in Figure 1i, where the output energy is represented by the area of a *V‐*‐*Q* loop.^[^ ^44^ ^]^ Note that a more detailed explanation for Figure 1i can be found in Note S2, Supporting Information.

Set *g* = 30 mm, and the situation of the reference electrode located at the upper of the primary electrode is also carried out in simulation because *g* is higher than *x*_max_. First, the *V*_OC_ and *Q*_SC_ with the reference electrode in different directions are calculated (Figure S4a‐b, Supporting Information), which rank from the largest to the smallest as: upper, upper right, right, below right, and below. The *V*_OCmax_ and *Q*_SCmax_ with the reference electrode located at the upper direction are both significantly higher than those at other directions (Figure S5a‐b, Supporting Information), where *V*_OCmax_ at upper is 1.59, 1.58, 1.56, and 1.52 times that at the other four directions, and *Q*_SC max_ at upper is 1.59, 1.59, 1.57 and 1.53 times that at the other four directions. Secondly, the maximum voltage (Figure S5a, Supporting Information), maximum current (Figure S5b, Supporting Information), and average power under different load resistors (Figure 1j and Figure S5c, Supporting Information) are also calculated. As shown in Figure 1k, the maximum average power at upper is 1.83, 1.72, 1.68, and 1.60 times higher than those at below, below right, right, and upper right, and the corresponding optimum load resistor is 1.23, 1.21, 1.20, and 1.18 times higher, respectively. The corresponding *V–Q* closed loops are presented in Figure 1l, further revealing the influence of the reference electrode's direction on the outputs. In addition, it can be seen from the results that the average power of the P‐CS‐SETENG when *g* = 30 mm is larger than that at *g* = 1 mm and the average power increases with the increase in *g*. These data indicate that, for the case of a larger gap (>*x*_max_), placing the reference electrode at the upper of the primary electrode is much more valuable than a horizontal bias, which yields obviously higher electrical outputs. The situation of placing the reference electrode directly at the upper of the primary electrode is similar to the FTENG, which has theoretically 100% charge‐transfer efficiency^[^ ^31^ ^]^ and better output performance by effectively avoiding the electrostatic shield effect.

Based on the above results, the optimization strategy regarding the reference electrode's location of P‐CS‐SETENGs can be analyzed as follows. On the one hand, for most of the practical wearable flexible devices, the gap is quite limited (usually not more than 10 mm) due to the restrained package space. Therefore, P‐CS‐SETENGs can be optimized with the largest possible gap, and the priority of the reference electrode’ locations are ranked as: the upper right, the right, the below right, and the below. On the other hand, if a relatively large gap (i.e., higher than the maximum separation displacement) between the primary and reference electrodes is achievable in practical applications, the reference electrode located directly at the upper of the primary electrode is the most favored, which can yield much higher electrical outputs.

Considering the fact that SETENGs have been mostly used for flexible/wearable applications where the reference electrode generally is encapsulated inside the device and the device thickness is small, in the rest sections of this paper, unless otherwise specified, the default case is with the primary and reference electrodes having a gap of 1 mm.

#### 2.1.2 The Effect of the Reference Electrode's Size

Besides the location of the reference electrode, the size parameters of the reference electrode, including the area and thickness, are also an essential design considerations for the output performance of P‐CS‐SETENGs.

**Figure 2:**
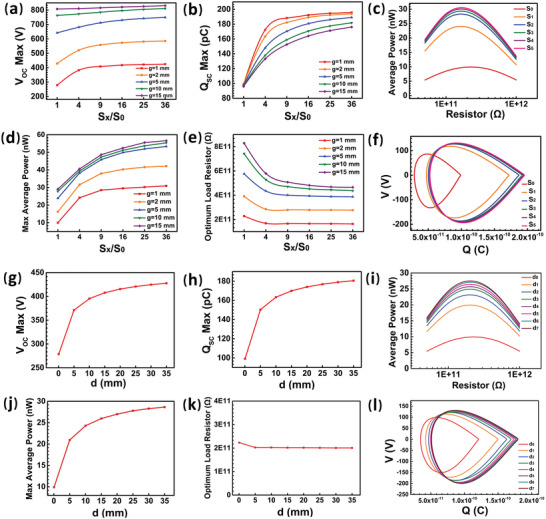
The effect of the reference electrode's size on the output performance of the P‐CS‐SETENG. (a,b,d,e) The a) V_OCmax_, b) Q_SCmax_, d) maximum average power, and e) optimum load resistor with different reference electrode areas at different gaps. c,f) The (c) average power and (f) *V–Q* plots under optimum load resistors with different reference electrode areas at *g* = 1 mm. g‐l) The (g) *V*_OCmax_, (h) *Q*_SCmax_, (i) average power, (j) maximum average power, (k) optimum load resistor, and (l) *V–Q* plots under optimum load resistors with different reference electrode thicknesses (*g* = 1 mm).

First, the output performance of P‐CS‐SETENGs with reference electrodes having different areas is calculated through the COMSOL software. The reference electrode's area (5 × 5 mm^2^) in the initial state is the same as the primary electrode's, which is defined as *S*_0_. The other reference electrode areas are defined as *S_x_*, where *S*_1_ = 10 × 10 mm^2^, *S*_2_ = 15 × 15 mm^2^, *S*_3_ = 20 × 20 mm^2^, *S*_4_ = 25 × 25 mm^2^, *S*_5_ = 30 × 30 mm^2^. The *V*_OC_ and *Q*_SC_ of P‐CS‐SETENGs with different reference electrode areas are shown in Figure S6a‐b, Supporting Information, and the corresponding *V*_OCmax_ and *Q*_SCmax_ are shown in **Figure** **2**a‐b. At *g* = 1 mm, the *V*_OCmax_ increases to 1.52 times and *Q*_SCmax_ increases to 1.97 times when the reference electrode area increases from 5 × 5 mm^2^ to 30 × 30 mm^2^. The growths of the *V*_OCmax_ and *Q*_SCmax_ with the *S_x_*/*S*_0_ are both first very fast, then slow, and eventually tend to saturate. These results suggest that modest increases in the reference electrode area are beneficial, while excessive increases are ineffective.

Such impact of the reference electrode area on the outputs can be intuitively interpreted by the distribution of electric field and potential (Figure S7a‐b, Supporting Information). Due to the physical properties of the parallel‐plate electric field, the farther away from the central axis of the primary and reference electrodes, the lower the potential. Therefore, when the area of the reference electrode is larger, it will have a lower potential, which results in a higher *V*_OC_. And the farther away from the central axis, the sparser the electric field lines, and consequently the slower the potential reduction. This explains why the growth of the V_OC_ becomes slower and slower and eventually saturates as the *S_x_*/*S*_0_ further increases.

Compared to the *V*_OC_, the *Q*_SC_ grows a bit faster as the *S_x_*/*S*_0_ increases. This can be explained by TENG's governing equation^[^ ^33^ ^]^ (named as *V–Q*–*x* relationship).

(2) $$V=-\frac{1}{C}Q+V_{OC}$$

Thus, under short‐circuit (SC) conditions, the fundamental relationship among the *Q_SC_*, *C*, and *V_OC_* can be obtained:

(3) $$Q_{SC}=V_{OC}\;C$$

where the capacitor *C* in the entire equivalent circuit is:

(4) $$C=C_{3}\;+\frac{C_{1}C_{2}}{C_{1}+C_{2}}$$

where *C*_1_ represents the capacitance formed between the contacting object and primary electrode, *C*_2_ represents the capacitance formed between the contacting object and reference electrode, *C*_3_ represents the capacitance formed between the primary electrode and reference electrode (Figure S8, Supporting Information). The equation of the capacitance of the square plate capacitor with different lengths of upper and lower plates is:

(5) $$C=\varepsilon_{0}\;\frac{ab}{h}$$

where *ε*_0_ represents the permittivity of vacuum, *a* represents the length of upper plates, *b* represents the length of lower plates and *h* represents the distance between the plates. The detailed derivation of Equation (5) can be found in Note S3, Supporting Information. As the area of the reference electrode increases, *C*_1_ is basically unchanged, while *C*_2_ and *C*_3_ increase, resulting in the increase in *C*. Combined with Equation (3), it well explains the faster growth of *Q_SC_* with the increasing *S_x_*/*S*_0_.

The average power of P‐CS‐SETENGs with the reference electrodes having different areas under different load resistors is shown in Figure 2c and Figure S6c, Supporting Information. The maximum average power of the P‐CS‐SETENG increases with the increasing area ratio *S_x_*/*S*_0_ (Figure 2d), while the optimum load resistor shifts to a lower value (Figure 2e). At *g* = 1 mm, the maximum average power increases to 3.03 times, and the optimum load resistor decreases to 72% when the area of the reference electrode is increased to 25 times. The improvements of the maximum average power and optimum load resistor by increasing the reference electrode area both tend to saturate gradually (Figure 2d,e), and the *V–Q* plots also verify these changing trends (Figure 2f).

In addition, the variation trends of the *V*_OC_, *Q*_SC_, maximum average power, and optimum load resistor with the changing *S_x_*/*S*_0_ when *g* = 2 mm, 5 mm, 10 mm, and 15 mm are similar to those when *g* = 1 mm. Under the same *S_x_*/*S*_0_, the *V*_OC_, maximum average power, and optimum load resistor all increase with the increasing g, whereas the Q_SC_ decreases (Figure 2a‐b,d‐e). Besides, with the increase in g, the critical value of *S_x_*/*S*_0_ for the saturation state of the maximum average power also increases (Figure 2d). More detailed data for the *V*_OC_, *Q*_SC_, and average power of P‐CS‐SETENGs with various reference electrode areas at different *g* can be found in Figure S9, Supporting Information. Therefore, for the device design in practice, the most suitable area is suggested to be set based on the consideration of the minimum critical area and maximum allowable area.

Next, the effect of the reference electrode thickness on the output performance of P‐CS‐SETENGs is investigated (*g* = 1 mm). The reference electrode thickness of 0.05 mm is defined as *d*_0_, and the other thicknesses are defined as *d_x_*, where *d*_1_ = 5 mm, *d*_2_ = 10 mm, *d*_3_ = 15 mm, *d*_4_ = 20 mm, *d*_5_ = 25 mm, *d*_6_ = 30 mm, *d*_7_ = 35 mm. The *V*_OC_ and *Q*_SC_ with different reference electrode thicknesses are shown in Figure S10a‐b, Supporting Information. It is found that the influence of the reference electrode thickness is similar to that of the reference electrode area. As shown in Figure 2g,h, the *V*_OCmax_ increases to 2.54 times and *Q*_SC max_ increases to 2.82 times when the thickness of the reference electrode increases from 0.05 mm to 35 mm. The growth rates of *V*_OCmax_ and *Q*_SCmax_ both slow down and approach their saturation values with the increasing reference electrode thickness. Such influence of the reference electrode thickness on the output performance can also be explained by potential distribution in the electrical field. As is shown in Figure S7c, Supporting Information, the farther away from the lower surface of the reference electrode, the lower the potential. Therefore, increasing the thickness of the reference electrode is beneficial for obtaining a lower potential and consequently increasing the *V*_OC_. Besides, the saturation phenomenon can also be explained by a similar mechanism to the previous analysis of the area factor.

The average power of P‐CS‐SETENGs with different reference electrode thicknesses under different load resistors is shown in Figure 2i and Figure S10c, Supporting Information. The maximum average power increases to 2.85 times when the thickness of the reference electrode is increased to 600 times (from 0.05 mm to 30 mm) (Figure 2j), and the optimum load resistor decreases slightly with the increase in the reference electrode thickness (Figure 2k). The change in the maximum average power of P‐CS‐SETENGs with different reference electrode thicknesses can also be expressed in terms of the area of *V–Q* closed loop in Figure 2l. It can be concluded from these results that an increase in the thickness of the reference electrode help enlarge the electrical outputs of P‐CS‐SETENGs but the enlargement rates all decrease with the increasing thickness.

**Figure 3:**
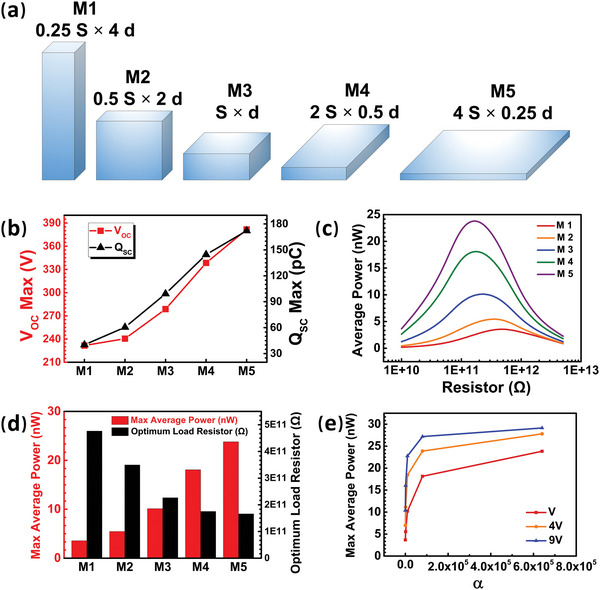
The effect of the area/thickness coefficient (*α* = *S*/*d* ^2^) of reference electrodes with the same volume on the output performance of the P‐CS‐SETENG. a) Schematic illustration showing reference electrodes with different *α*. b‐d) The (b) *V*_OCmax_ and *Q*_SCmax_, (c) average power, and (d) maximum average power and optimum load resistor with reference electrodes of different *α*. e) The relationships between the maximum average power and *α* under different reference electrode volumes. Note that *g* = 1 mm.

Actually, for many practical systems, the volume constraint of the cuboid reference electrode is generally a fixed value. Therefore, increasing the area and increasing the thickness are often in conflict, and it is found that maximizing the output performance depends on the optimized coefficient *α* = *S*/*d* ^2^. As shown in **Figure** **3**a, Models M1∼M5 are designed as 0.25 S × 4 d, 0.5 S × 2 d, S × d, 2 S × 0.5 d, and 4 S × 0.25 d, where the corresponding *α* are 1/16, 1/4, 1, 4, and 16. As shown in Figure 3b, from Model M1 to M5, as *α* becomes larger, both the *V*_OCmax_ and *Q*_SCmax_ increase sequentially (e.g., the *V*_OCmax_ and *Q*_SCmax_ of M5 are 1.65 times and 4.28 times that of M1, respectively). Furthermore, the maximum average power of M5 is 6.67 times higher than that of M1, while the optimum load resistor of Model 5 is only 35% that of M1(Figure 3c‐d). The corresponding more detailed data can be found in Figure S11, Supporting Information. It can be concluded that the P‐CS‐SETENG with a reference electrode of a larger *α* (which means smaller thickness and larger area) can obtain higher maximum average power and lower corresponding optimum load resistor simultaneously under a constant volume constraint. In other words, the effect of the reference electrode area on the output performance of P‐CS‐SETENGs is more dominant than that of the reference electrode thickness.

The dominance of the area's influence is determined by the distribution of the electric field and potential. The decrease in potential in the horizontal direction along the reference electrode's side is much sharper than that in the vertical direction along the thickness (Figure S7, Supporting Information). Therefore, compared with the increase in thickness, the increase in the reference electrode's area is more effective to achieve a lower potential relative to the primary electrode and thereby a larger *V*_OC_. Apart from that, the increase in the area also contributes to an increase in the capacitance and short‐circuit transferred charge *Q*_SC_. These two aspects jointly determine that the area of the reference electrode is a more dominant factor for improving the output performance than the thickness.

Furthermore, the relationships between the maximum average power and *α* under different reference electrode volumes reveal that the maximum average power under the same *α* increases when the volume of the reference electrode increases (from *V* to 9 V, *V* is a certain volume), and approaches its saturation value as *α* rises with the same volume (Figure 3e). In addition, the maximum average power of the P‐CS‐SETENG with a larger volume of the reference electrode saturates at a smaller *α*, which indicates that the above‐mentioned reference electrode surface area's dominant influence on the outputs weakens with the increase in the reference electrode's volume. Note that more detailed data for the output performance with reference electrode volumes of 4 V and 9 V can be found in Figure S12, Supporting Information.

Based on the above results, the optimization strategy concerning the reference electrode's size of P‐CS‐SETENGs can be obtained as follows. First, when the reference electrode is constrained by area or thickness, designing the area and thickness parameters with their upper bound constraint values or simulated critical saturation values is beneficial for achieving the highest maximum average power and the lowest optimum load resistor. Second, when the reference electrode is constrained by the volume, a larger area and thinner thickness for the reference electrode is preferred for acquiring the optimal output performance.

#### 2.1.3 The Effect of the Reference Electrode's Shape

In addition to the reference electrode's location and size, the effect of the reference electrode's shape on the output performance of P‐CS‐SETENGs is also studied. Under a fixed volume, the reference electrodes with different shapes for the finite element calculation of simulation was carried out and studied thoroughly. It is found that among various reference shapes such as triangular pyramid, rectangular pyramid, cone, triangular prism, quadrangular, cylinder, and sphere, the reference electrode shaped in triangular pyramid contributes to the highest output performance whereas the reference electrode shaped in sphere possesses the lowest maximum average power. The detailed data and analysis can be found in Figures S13‐S15, Supporting Information, and Note S4, Supporting Information.

### 2.2 P‐S‐SETENG Working by Sliding Motion

Besides the vertical contact/separation mode, SETENGs can also work based on in‐plane horizontal charge separation, which is the sliding‐mode SETENGs.^[^ ^32^ ^]^ Through the simulation software COMSOL, the P‐S‐SETENG model was constructed, which consists of three parts: the contacting object, the primary electrode, and the reference electrode. The contacting object slides along the upper surface of the primary electrode.

The primary electrode and reference electrode are fixed while the contacting object can laterally slide. More details for the theoretical simulation model of P‐S‐SETENGs can be found in Note S5, Supporting Information.

#### 2.2.1 The Effect of the Reference Electrode's Location

**Figure 4:**
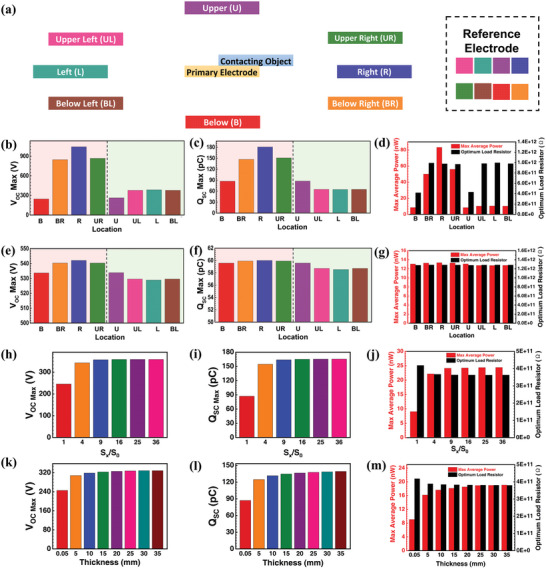
The reference electrode's influence on the output performance of the P‐S‐SETENG. a) Schematic illustration showing the reference electrode at different locations. b‐d) The (b) *V*_OCmax_, (c) *Q*_SCmax_, and (d) maximum average power and optimum load resistor under different reference electrode locations at *g* = 1 mm. e‐g) The (e) *V*_OCmax_, (f) *Q*_SCmax_, and (g) maximum average power and optimum load resistor under different reference electrode locations at *g* = 30 mm. h‐j) The (h) *V*_OCmax_, (i) *Q*_SCmax_, and (j) maximum average power and optimum load resistor under different reference electrode areas at *g* = 1 mm. k‐m) The (k) *V*_OCmax_, (l) *Q*_SCmax_, and (m) maximum average power and optimum load resistor under different reference electrode thicknesses at *g* = 1 mm.

First, at *g* = 1 mm, the reference electrode is placed in different locations to investigate its influence on the output performance of P‐S‐SETENGs. The horizontal sliding separation direction of the contacting object is defined as the right side. Thus, the directions of the reference electrode includes below, below right, right, upper right, upper, upper left, left, and below left (**Figure** **4**a). The *V*_OCmax_ and *Q*_SCmax_ of the P‐S‐SETENG by *x*_max_ = 5 mm when the reference electrode located at different directions are summarized in Figure 4b,c. It can be seen that the reference electrode on the right side can maximize the *V*_OC_ and *Q*_SC_ of the P‐S‐SETENG, followed by the below right and upper right, and then the others. The *V*_OCmax_ and *Q*_SCmax_ at right are 4.25 times and 2.07 times higher than those at below, respectively. Furthermore, the maximum average power and optimum load resistor are summarized in Figure 4d. It can be found that when the reference electrode is located at the right of the primary electrode, the P‐S‐SETENG can obtain the highest maximum average power, which is 9.78 times higher than that at below. The corresponding more detailed data can be found in Figure S16, Supporting Information.

The above results can be ascribed to the fact that the equipotential lines of the electric field distribute from the largest to the smallest from the primary electrode to the contacting object (Figure S17, Supporting Information), and the reference electrode placed near the contacting object will obtain a lower potential accordingly, leading to a higher *V*_OC_ between the primary electrode and reference electrode. Therefore, when the reference electrode is located to the right of the primary electrode, the maximum output performance is obtained. In fact, the P‐S‐SETENG with the reference electrode located at right is in structure similar to the sliding‐mode FTENG, whose charge‐transfer efficiency can reach 100% theoretically.^[^ ^31^ ^]^

Then, the influence of the reference electrode's location on the P‐S‐SETENG's performance at *g* = 30 mm is investigated. As shown in Figure 4e‐g, unlike at *g* = 1 mm, there is no significant difference in the *V*_OCmax_, *Q*_SCmax_, maximum average power, and optimum load resistor when the reference electrode is in different directions. For example, the maximum average power at right is the highest while at left is the smallest, with only ≈1.05 times higher at right than at left. It can be concluded that as *g* becomes larger, the reference electrode's location has a much weaker influence on the output performance of the P‐S‐SETENG, which is consistent with the results obtained from the P‐CS‐SETENG discussed earlier. Furthermore, it is found that the values of the electrical outputs under different reference electrode locations at *g* = 30 mm are all between the highest and lowest values in the case of *g* = 1 mm. The corresponding more detailed data about the *V*_OC_, *Q*_SC_, average power, and *V–Q* curves can be found in Figure S18, Supporting Information.

Additionally, it is noticed from Figure 4b‐g that the alteration in the reference electrode locations on the right side has a bigger influence on the performance compared with the left side, especially when g is small. This phenomenon is mainly because that the contacting object slides over the right side. The strength of the electric field of the contacting object on the right side is higher than on the left (Figure S17, Supporting Information); consequently, as the contacting object slides over the right side, there will be larger potential differences between the primary and reference electrodes when there's alteration in the reference electrode's locations at the right side compared with at the left side. Hence the alteration in the reference electrode locations at the side where the contacting object slides over presents a stronger influence on the performance.

Based on the above results, the optimization strategies for P‐S‐SETENGs’ reference electrode location can be acquired as follows. First, the reference electrode is suggested to be distributed staggered with the primary electrode, especially on the side where the sliding motion covers. Second, if the reference electrode has to be placed directly below the primary electrode, the gap between the reference and primary electrodes is preferred to be set as large as possible within the device size constraint.

#### 2.2.2 The Effect of the Reference Electrode's Size

The impact of the reference electrode's size, including the area and thickness, on the output performance of the P‐S‐SETENG is also examined. The parameters set in the simulation are the same as those for the P‐CS‐SETENG in Section 2.1.2 (*g* = 1 mm). As shown in Figure 4h,i, the *V*_OCmax_ and *Q*_SCmax_ increase with the enlarging reference electrode area, both saturate as the area further increases. As shown in Figure 4j, the maximum average power increases with the enlarging reference electrode area at a decreasing rate, while the optimum load resistor shifts to a lower value. As the area increases to 9 times, the *V*_OCmax_, *Q*_SCmax,_ and maximum average power increase to 1.45 times, 1.88 times, and 2.66 times, respectively, and the optimum load resistor decreases to 87%, all saturating when the area further increases. As for practical applications, since the enlargement of the reference electrode area means higher cost, the most suitable reference electrode area can be determined by considering the trade‐off between the high outputs and low cost. The corresponding more detailed data can be found in Figure S19, Supporting Information.

Similarly, with the increasing reference electrode thickness, the *V*_OCmax_, *Q*_Scmax,_ and maximum average power increase while the optimum load resistor reduces, all at decreasing rates (Figure 4k,l,m). When the thickness of the reference electrode increases from 0.05 mm to 25 mm, the *V*_OCmax_, *Q*_SCmax_, and maximum average power increase to 1.33 times, 1.58 times, and 2.11 times, respectively; while the optimum load resistor decreases to 91%. With the thickness further increases, there are trivial improvements in the outputs. Therefore, for practical device designs, an optimal thickness value can also be determined. The corresponding more detailed data can be found in Figure S20, Supporting Information.

### 2.3 Extending Studies

Apart from SETENGs structured with the working (primary) electrode serving as the triboelectric layer, SETENGs structured with a dielectric layer attached onto the primary electrode, namely the D‐SETENGs, are also widely applied. The basic finite element model of D‐SETENGs is shown in Figure S21, Supporting Information, and its only difference in structure from P‐SETENGs is that one triboelectric layer is the dielectric layer attached on top of the primary electrode instead of the primary electrode. The D‐SETENGs can be also divided into two types like P‐SETENGs: the contact/separation mode (D‐CS‐SETENG) and sliding mode (D‐S‐SETENG).

Further systematical simulation results and theoretical analysis reveal that the existence of the dielectric layer does not affect the basic influences of the reference electrode on the SETENGs’ electrical outputs. Therefore, the design optimization strategies proposed above for the P‐CS‐SETENG and P‐S‐SETENG can also be applied to the D‐CS‐SETENG and D‐S‐SETENG, respectively. The corresponding detailed data and discussions can be found in Figures S22–S26 and Note S6, Supporting Information.

In addition, considering that SETENGs are widely applied for flexible/wearable electronics where the SETENG devices often encounter bending strain. The influence of the reference electrode's bending state on the SETENGs’ performance was also studied. It is found that if the reference electrode is under bending and the working part is in the original state (*g* = 1 mm, contact/separation mode), the *V*_OC_, *Q*_SC,_ and maximum average power all slightly increase as the bending angle rises (Figures S27–S29, Supporting Information). If both the reference electrode and working part are under bending (*g* = 1 mm, contact/separation mode), as the bending angle rises, the V_OC_, Q_SC_ slightly decrease while the maximum average power first enhances and then declines (Figures S30–S32, Supporting Information).

## 3 Experimental Verification for the Reference Electrode's Influence on the Performance of SETENGs

On the basis of the conclusions and structural optimization strategies derived from the above simulation results, we carried out experiments to verify the primary ones. The details of the materials, device fabrication, and measurement setup for the experimental verification can be found in Figure S33, Supporting Information and the Experimental Section.

**Figure 5:**
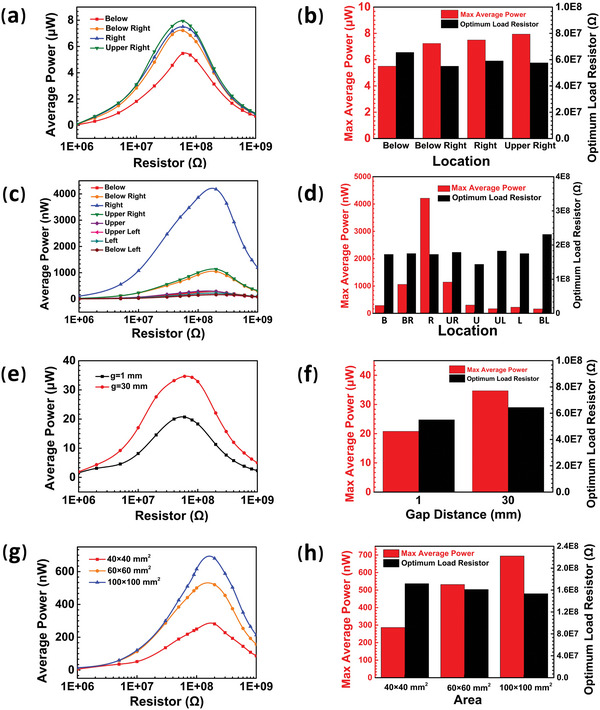
Experimental verification for the reference electrode's impact on the performance. a‐b) The (a) average power and (b) maximum average power and optimum load resistor were measured from P‐CS‐SETENGs with reference electrodes at different locations. c‐d) The (c) average power and (d) maximum average power and optimum load resistor were measured from P‐S‐SETENGs with reference electrodes at different locations. e‐f) The (e) average power and (f) maximum average power and optimum load resistor were measured from P‐CS‐SETENGs with the reference electrode placed directly below the primary electrode at different gaps. g‐h) The (g) average power and (h) maximum average power and optimum load resistor were measured from P‐CS‐SETENGs with different reference electrode areas at *g* = 1 mm.

First, the influence of the reference electrode's location on the output performance of SETENGs, both the vertical contact/separation mode and horizontal sliding mode, is verified. The experimental data shown in **Figure** **5**a,b show that the average powers under different load resistors including the maximum average power of the fabricated P‐CS‐SETENG have values ranking from high to low when the reference electrode is located at oblique upper, right, below right, and below. The measured maximum average power of the P‐CS‐SETENG with the reference electrode at oblique upper is 1.44 times higher than that measured at below. As presented in Figure 5c,d, the fabricated P‐S‐SETENG achieves the highest performance when the reference electrode is at right (i.e., parallel to the primary electrode and in a direction where the freely moving contacting layer can slide over). The measured maximum average power of the P‐S‐SETENG when the reference electrode locates at right is 14.71 times higher than that at below (Figure 5d). These experimental data are in agreement with the theoretical simulation results, demonstrating the established basic theoretical models are fundamentally reliable.

Secondly, the effect of the gap between the primary and reference electrodes on the performance is verified. The theoretical simulation results suggest that the electrical outputs of the P‐CS‐SETENG are improved with the increase in the gap. As shown in Figure 5e, the increase in the gap (from 1 mm to 30 mm) leads to a conspicuous increase in the average power of the fabricated P‐CS‐SETENG. The maximum average power measured at *g* = 30 mm is 1.67 times higher than that at *g* = 1 mm while the optimum load resistor is also higher at *g* = 30 mm than that at *g* = 1 mm (Figure 5f), which are all consistent with the theoretical simulation results.

Finally, the effect of the reference electrode's area on the output performance is certified to be consistent with the theoretical results. The theoretical simulation results indicate that the output performance of the SETENGs increases with the increase in the reference electrode area. It can be seen from Figure 5g,h that the measured average power of the fabricated P‐S‐SETENG enhances when the area of the reference electrode enlarges (*g* = 1 mm). The maximum average power of the P‐S‐SETENG with a reference electrode area of 60×60 mm^2^ is 1.86 times higher than that of the P‐S‐SETENG with a reference electrode area of 40×40 mm^2^, and the maximum average power of the P‐S‐SETENG with a reference electrode area of 100×100 mm^2^ is 2.42 times higher than that of the P‐S‐SETENG with a reference electrode area of 40×40 mm^2^. The corresponding optimum load resistor decreases with the increasing reference electrode area.

Note that there are differences in the specific values of the electrical outputs obtained from the experiments and simulation, which are mainly because of the inevitable discrepancy in the surface charge density between the experiments and simulation and unavoidable measurement errors during the experiments. Although the specific values have differences, the changing trends acquired from the experiments and simulation are basically the same. The above verification experiments reveal that the experimental results are basically consistent with the theoretical simulation results, which demonstrates the reliability of the theoretical simulation results as the fundamental basis. These experimental results also prove that our theoretical simulations can provide effective fundamental guidance for the optimization of SETENGs’ output performance.

## 4 Conclusion

In summary, we have systematically and comprehensively investigated the effect of the reference electrode on the output performance of SETENGs through theoretical simulation and experimental verification. 1) As for the reference electrode's direction, SETENGs have the lowest performance when the reference electrode is located at perpendicularly below the primary electrode. Higher electrical outputs are obtained when the reference electrode is located at upper or oblique upper of the primary electrode for vertical‐contact/separation‐mode SETENGs and at parallel to the primary electrode where the sliding motion covers for sliding‐mode SETENGs compared with at other directions, respectively. But such influences wane as the gap between the primary and reference electrodes increases. 2) The electrical outputs of SETENGs enhance at a decreasing rate with the increasing reference electrode area, thickness, and volume. The reference electrode area plays a heavier role in the improved performance than the thickness when the reference electrode's volume is fixed. 3) As for the reference electrode's shape, the reference electrode in triangular‐pyramid shape helps achieve higher electrical outputs than in other shapes whereas shaped in sphere brings about inferior performance. 4) The attachment of a dielectric layer onto the primary electrode leads to changes in the magnitudes of the electrical outputs but does not alter the changing trends of the performance by the reference electrode.

To achieve optimum output performance in practical applications, within the constraint of device space, the optimization strategies of the reference electrode design can be summarized as follows. 1) The reference electrode position is suggested to be staggered with the primary electrode; specifically, if possible, placed at upper or oblique upper for vertical‐contact/separation‐mode SETENGs and parallel to the primary electrode where the sliding motion covers for sliding‐mode SETENGs. 2) It is preferred to increase the reference electrode's size (area or thickness, especially the area), as large as possible within the size constraints. (3) It is favorable to make the reference electrode into triangle shapes (especially triangular pyramid) with a larger area/thickness ratio. Note that brief schematic illustrations for the main optimization strategies are exhibited in Figure S34, Supporting Information.

In addition, trade‐offs between the performance and other factors such as fabrication cost may need to be assessed in practical applications, and system errors of the testing measurements may also need to be considered when evaluating the electrical outputs of SETENGs. This paper provides important fundamental guidance for the development of TENGs and offers valuable insights for the design of new energy‐harvesting and sensing devices.

## 5 Experimental Section

### Materials and Instrumentation

1) The PTFE film, copper sheet, and acrylic plate were purchased from Taobao. 2) The output voltages under different resistor loads were measured by Keithley 6514 System Electrometer. 3) The contact and separation process of the contacting object was driven by a LinMot E1200‐GP‐UC linear motor. 4) The acrylic sheet was cut to appropriate sizes by a 4060 laser machine. 5) The device structure and position were adjusted by a GM‐1711 M lifting platform. 6) The load resistor was adjusted through a MC‐21‐B adjustable resistance box.

### Structures of the SETENGs

1) The copper sheets with a size of 10 cm × 10 cm × 1 mm were chosen as the primary electrode and reference electrodes of the P‐CS‐SETENGs, the PTFE film with a size of 10 cm × 10 cm × 0.2 mm was chosen as the contacting layer of the P‐CS‐SETENGs. 2) The copper sheets with a size of 4 cm × 4 cm × 0.5 mm were chosen as the primary electrode and reference electrodes of the P‐S‐SETENG, the PTFE film with a size of 4 cm × 4 cm × 0.2 mm was chosen as the contacting layer of the P‐S‐SETENG.

### Fabrication and Measurement setup of the SETENGs

1) The PTFE film was attached to an acrylic plate, and the primary electrode and reference electrode were separated with an acrylic plate of appropriate sizes. 2) The primary electrode and reference electrode were mounted onto the upper and bottom surfaces of an acrylic plate, respectively. 3) The PTFE film with an acrylic plate was fixed onto the moving end of a linear motor, and the electrodes were mounted onto a fixed end along the moving direction of the linear motor (Figure S27, Supporting Information). 4) The copper plates were connected to the adjustable resistance box through copper wires. 5) The resistance box was connected to the electrical measuring system through private wires.

## Conflict of Interest

The authors declare no conflict of interest.

## Author Contributions

F.Y. conceived the idea. Z.C., J.Z., D.Z., and X.L. performed the simulations and experiments. Z.C., F.Y., and D.K. graphed and analyzed the data. F.Y., Z.C., and D.K. wrote the paper. All authors discussed the results and commented on the manuscript.

## Supporting information

Supporting Information

## Data Availability

The data that support the findings of this study are available from the corresponding author upon reasonable request.
