# Supplementary material for: Influence of the Reference Electrode on the Performance of Single‐Electrode Triboelectric Nanogenerators and the Optimization Strategies
Source: Adv Sci (Weinh). 2023 Apr 23;10(17):2206950. doi: 10.1002/advs.202206950 (PMC10265061; doi:10.1002/advs.202206950)

ADVANCED SCIENCE
Open Access

Supporting Information

for *Adv. Sci.*, DOI 10.1002/advs.202206950

Influence of the Reference Electrode on the Performance of Single-Electrode Triboelectric Nanogenerators and the Optimization Strategies

Zetong Chen, Keren Dai, Jiaxiang Chen, Jingting Zhuo, Danna Zhao, Rui Ma, Xujing Zhang, Xubiao Li, Xiaofeng Wang*, Guowei Yang and Fang Yi**

Supporting Information

Influence of the reference electrode on the performance of single-electrode triboelectric nanogenerators and the optimization strategies

Zetong Chen, Keren Dai*, Jiaxiang Chen, Jingting Zhuo, Danna Zhao, Rui Ma, Xujing Zhang, Xubiao Li, Xiaofeng Wang*, Guowei Yang, and Fang Yi*

Z. Chen, J. Chen, J. Zhuo, D. Zhao, R. Ma, X. Zhang, X. Li, Prof. G. Yang, Prof. F. Yi

School of Materials Science and Engineering

Nanotechnology Research Center

Guangzhou Key Laboratory of Flexible Electronic Materials and Wearable Devices

State Key Laboratory of Optoelectronic Materials and Technologies

Sun Yat-sen University

Guangzhou, 510275, P. R. China

E-mail: yifang@mail.sysu.edu.cn (F. Yi)

Dr. K. Dai

School of Mechanical Engineering

Nanjing University of Science and Technology

Nanjing 210094, P. R. China

E-mail: dkr@njust.edu.cn (K. Dai)

Prof. X. Wang

Department of Precision Instrument

Beijing Advanced Innovation Center for Integrated Circuits

Tsinghua University

Beijing, 100084, P. R. China.

E-mail: xfw@tsinghua.edu.cn (X. Wang)

Note S1. Detailed information and parameter settings for the theoretical simulation of the P-CS-SETENG (contact/separation-mode SETENG structured with the primary electrode serving as the triboelectric layer).

The theoretical simulation model consisting of the contacting object (5×5×0.1 mm^3), primary electrode (5×5×0.05 mm^3) and reference electrode (5×5×0.05 mm^3) was constructed through the simulation software COMSOL. The material with a relative permittivity of 2 is employed as the contacting object. The material selected for the primary electrode and the reference electrode is copper. Furthermore, since the electric field in the electrode is zero everywhere and charges only remain on the surface of the electrode, the interior of electrode is not considered in the calculation of the electrostatic field. The contacting object and primary electrode are stacked face to face to form the paired triboelectric layers, and the reference electrode locates at the below of the primary electrode with a varying gap distance. The contacting object moves along the vertical direction, and the separation distance between the two triboelectric layers is defined as x. The maximum separation distance (x_{max}) between the contacting object and primary electrode is set as 28 mm. This whole geometry structure is surrounded by a sphere with a radius of 0.05 m, and the interior of the sphere is filled with a material with a relative permittivity of 1, which is used to mimic the air environment. The outermost layer of the sphere is set as infinite element field, which is used to simulate that the simulation model is placed in an area without boundary. The triboelectric charge densities on the bottom surface of the contacting object are assigned as -8μC m^{-2}, and an equal amount of positive charges (5×5×8×10^{-6} μC) are injected to the surface of the primary electrode through contact electrification. The voltage between the two electrodes under open-circuit conditions is defined as V_{OC}. With Q defined as the transferred charges from the primary electrode to the reference electrode, the short-circuit transferred charge is defined as Q_{SC} when the voltage is

equal to zero at the short-circuit condition. The parameters settings used in this simulation model are listed in Table S1.

Table S1. Utilized parameters in the calculation of the P-CS-SETENG's output characteristics.

Permittivity of the contacting object<br>Permittivity of air	ε_1= 2<br>ε_2= 1
Size of the contacting object	w= 5 mm, l= 5 mm, d_d= 0.1 mm
Material of the electrodes	Copper
Size of the primary electrode	w= 5 mm, l= 5 mm, d= 0.05 mm
Triboelectric charge surface density σ	8 μC m^{-2}
Maximum separation distance x_{max}	28 mm
Average velocity v	14 mm/s

Note S2. Detailed explanation for the maximum average power represented by the area of the corresponding V-Q closed loop.

The electrical outputs of TENGs from a continuous periodic mechanical motion are also periodically time-dependent. The corresponding average output power P_a that is associated with the load resistor can be applied to characterize the performance of the TENG. The output energy per cycle E derived from a certain period of time T can be calculated as:

$$E = P_a T = \int_0^T VIdt = \int_{t=0}^{t=T} VdQ = \oint VdQ \quad \text{(S1)}$$

The plot of the output voltage V against the transferred charges Q can represent the energy output of the TENG. Since the TENG has steady-state periodic output signals, the V-Q curve is a closed loop. The output energy per cycle E calculated from Equation S1 can be interpreted as the encircled area of V-Q closed loop. The maximum average power P_a uner the optimum load resistor can be acquired from dividing the output energy per cycle E by a certain period of time T:

$$P_a = \frac{E}{T} = \frac{\oint VdQ}{T} \propto \oint VdQ \quad \text{(S2)}$$

Hence, the maximum average power under the optimum load resistor can be represented by the area of the corresponding V-Q closed loop.

Note S3. Detailed derivation of the capacitance equation for square-plate capacitors with different lengths of upper and lower plates.

As shown in Figure S35a, the length of the upper plate is represented as a, the length of the lower plate is represented as b and the distance between the plates is represented as h. Two square plates with a distance of dh can form a capacitor, as given by:

$$dC = \varepsilon_0 \frac{4x^2}{dh} \tag{S3}$$

As shown in Figure S35b, according to the property that the corresponding sides of similar triangles are proportional, the following formula can be obtained:

$$\frac{dx}{b/2} = \frac{dh}{h+y} \tag{S4}$$

$$\frac{y}{y+h} = \frac{a/2}{b/2} = \frac{a}{b} \tag{S5}$$

Equations S5 can then be deduced as:

$$y = \frac{ah}{b-a} \tag{S6}$$

Combining Equations S4 and S6, we can obtain the equation as shown below.

$$dh = \frac{2(h+y)}{b} dx = \frac{2\left(h+\frac{ah}{b-a}\right)}{b} dx = \frac{2h(b-a)+2ah}{b(b-a)} dx = \frac{2h}{(b-a)} dx \tag{S7}$$

Thus, substituting Equations S7 into Equation S3, dC can be given as:

$$dC = \varepsilon_0 \frac{4x^2}{dh} = \varepsilon_0 \frac{4x^2}{\frac{2h}{(b-a)}dx} = 2\varepsilon_0 \frac{(b-a)}{h} \frac{x^2}{dx} \tag{S8}$$

Then the above capacitors are connected in series to obtain the total capacitance as follows:

$$C = \frac{1}{\int_{a/2}^{b/2} \frac{1}{dC}} = \frac{1}{\int_{a/2}^{b/2} \frac{1}{\frac{2\varepsilon_0 (b-a) x^2}{hdx}}} = \frac{1}{\int_{a/2}^{b/2} \frac{hdx}{2\varepsilon_0 (b-a) x^2}} = \frac{1}{\frac{h}{2\varepsilon_0 (b-a)} \int_{a/2}^{b/2} \frac{dx}{x^2}} = \frac{1}{\frac{h}{2\varepsilon_0 (b-a)} \left(\frac{1}{a/2} - \frac{1}{b/2}\right)} = \frac{\varepsilon_0 (b-a) ab}{h(b-a)} =$$

$$\varepsilon_0 \frac{ab}{h} \tag{S9}$$

Note S4. The effect of the reference electrode's shape on the output performance of P-CS-SETENGs.

The reference electrodes with different shapes are categorized into two cases. In the first case, the reference electrodes with different shapes have the same volume and the same thickness; while in the second case, the reference electrodes with different shapes have the same volume and the same area. The shape of the reference electrode can be specifically divided into the following types: triangular pyramid, rectangular pyramid, cone, triangular prism, quadrangular, cylinder, and sphere (Figure S13).

Firstly, the V_{OC} and Q_{SC} of P-CS-SETENGs with the first-case reference electrodes increase with the increase in the separation distance (Figure S14a-b). And the V_{OCmax} and Q_{SCmax} calculated at the maximum separation distance are shown in Figure S14c. The V_{OCmax} values of P-CS-SETENGs arranged in a descending order with the first-case reference electrodes are: pyramid, sphere, and prism. For instance, the V_{OCmax} of the device with the reference electrode shaped in triangular pyramid is 1.28 times higher than that of the device with the reference electrode shaped in triangular prism, and 1.23 times higher than that of the device with the reference electrode shaped in sphere. As for the Q_{SC}, the P-CS-SETENG with the pyramid-shaped reference electrode has the highest Q_{SC}, followed by the prism-shaped, and the device with the sphere-shaped reference electrode has the smallest Q_{SC}. For instance, the Q_{SCmax} of the device with the triangular-pyramid-shaped reference electrode is 1.59 times higher than that of the device with the triangular-prism-shaped reference electrode, and 6.12 times higher than that of the device with the sphere-shaped reference electrode. Furthermore, Figure S14d shows the average power of P-CS-SETENGs with reference electrodes in different shapes under various load resistors, it can be seen that the maximum average power of the device with the pyramid-shaped reference electrode is the highest, the prism-shaped is the second highest, and the sphere-shaped is the smallest, while their corresponding optimum load resistors are just in the

opposite order (Figure S14e). The maximum average power of the device with the triangular-pyramid-shaped reference electrode is 7.4 times higher than that of the device with the sphere-shaped reference electrode, while its optimum load resistor is only 18% that of the device with the sphere-shaped reference electrode. Note that the V-Q plot corresponding to each maximum average power for P-CS-SETENGs can be found in Figure S14f.

Secondly, P-CS-SETENGs with the second-case reference electrodes are constructed to study the effect of the reference electrode's shape on the output performance. The V_{OC} and Q_{SC} of the device with the pyramid-shaped reference electrode is slightly higher than that of the device with the prism-shaped reference electrode (Figure S15a-b). For example, the V_{OCmax} of the device with the triangular-pyramid-shaped reference electrode is 1.07 times higher than that of the device with the triangular-prism-shaped reference electrode, and the Q_{SCmax} is 1.01 times higher than that of the device with the triangular-prism-shaped reference electrode (Figure S15c). Besides, Figure S15d shows the average power of the device with reference electrodes in different shape under different load resistors, and the maximum average power and optimum load resistor are calculated in Figure S15e. The maximum average power values rank from high to low of the P-CS-SETENGs with the second-case reference electrodes are: pyramid, prism and sphere. For instance, the maximum average power of the device with the triangular-pyramid-shaped reference electrode is 8.11% higher than that of the device with the triangular-prism-shaped reference electrode. As for the optimum load resistor, it is arranged from large to small as follows: sphere, pyramid, prism. The optimum load resistor of the device with the triangular-pyramid-shaped reference electrode is 8.5% higher than that of device with the triangular-prism-shaped reference electrode. Note that the V-Q plot corresponding to each maximum average power can be found in Figure 15f.

It can be found that compared with the device with the first-case reference electrode, the enhancement of the output performance of the device with the second-case reference electrode

by the pyramid-shaped reference electrode is somewhat weakened. This can be attributed to the fact that the dominant factor of the first-case reference electrode is the area but of the second-case reference electrode is the thickness. And it has been revealed in the previous section in this work that the area plays a more dominant role in the output performance than the thickness.

In summary, the output performance of P-CS-SETENGs with the pyramid-shaped reference electrode is higher than that with the prism-shaped and sphere-shaped reference electrodes, which may be attributed to reason that the point effect exists at the tip of the pyramid reference electrode. Compared with the smooth part, the surface charge density at the tip is larger, which makes the change of electric field near the tip stronger. However, other factors should also be considered in analyzing the effect of the reference electrode's shape on the output performance. For example, it is often inevitable that the area factor and thickness factor should be taken into account. Based on the above simulation data analysis, if considering the shape factor alone, the triangular-pyramid shape of the reference electrode is the most favorable shape to improve the output performance of the device. Therefore, to achieve high electrical outputs, it is preferred for the reference electrode to shape in triangular pyramid.

Note S5. Detailed information and parameters settings for the theoretical simulation of P-S-SETENGs (sliding-mode SETENGs structured with the primary electrode serving as the triboelectric layer).

The theoretical simulation model composed of the contacting object ($5 \times 5 \times 0.1$ mm^3), primary electrode ($5 \times 5 \times 0.05$ mm^3) and reference electrode ($5 \times 5 \times 0.05$ mm^3) was constructed through the simulation software COMSOL. The material with a relative permittivity of 2 was applied as the contacting object. The material selected for the primary electrode and the reference electrode is copper. Moreover, since the electric field in the electrode is zero everywhere and charge only remains on the surface of the electrode, the interior of electrode is not considered in the calculation of the electrostatic field. The reference electrode is located at the below of primary electrode with a gap distance of 1 mm/30 mm. The contacting object is attached to the upper surface of the primary electrode. The primary electrode and reference electrode are fixed while the contacting object can slide along the lateral direction. The maximum separation distance (x_{max}) between the contacting object and primary electrode is set to be 5 mm. This whole geometry structure is surrounded by a sphere with a radius of 0.05 m; and the interior of the sphere is filled with a material with a relative permittivity of 1, which is used to mimic the air environment. The outermost layer of the sphere is set as infinite element field, which is used to simulate that the simulation model is placed in an area without boundary. The triboelectric charge densities on the the surfaces of the primary electrode and contacting object are assigned as $\pm 8 \mu C\ m^{-2}$. The voltage between the two electrodes under the open-circuit condition is defined as V_{OC}. With Q defined as the transferred charges from the primary electrode to the reference electrode, the short-circuit transferred charges was defined as Q_{SC} when the voltage equals to zero at the short-circuit condition. The parameters settings used in this simulation model are listed in Table S2.

Table S2. Utilized parameters in the calculation of the P-S-SETENG's output characteristics.

The permittivity of contacting object	ε_1= 2
The permittivity of air	ε_2= 1
Size of contacting object	w= 5 mm, l= 5 mm, d_d= 0.1 mm
Material of electrode	Copper
Size of electrode	w= 5 mm, l= 5 mm, d= 0.05 mm
Gap distance between electrodes g	1 mm and 30 mm
Triboelectric charge surface density σ	8 μC m^{-2}
Maximum separation distance x_{max}	5 mm
Average velocity v	14 mm/s

Note S6. The reference electrode's influence on the output performance of D-SETENGs (SETENGs structured with a dielectric layer attached onto the primary electrode).

Other than SETENGs structured with the primary electrode serving as the triboelectric layer, SETENGs structured with a dielectric layer attached onto the working (primary) electrode (D-SETENGs) are also widely applied. The basic finite element model of D-SETENGs is shown in Figure S21, and the only difference in structure from the P-SETENGs is that a dielectric layer on top of the primary electrode is utilized as the triboelectric layer. The D-SETENGs can also be devided into two types, including the contact-separation mode (D-CS-SETENG) and sliding mode (D-S-SETENG).

Analysis and optimization for D-CS-SETENGs.

The effect of the reference electrode location on the output performance of the D-CS-SETENG at g = 1 mm is studied firstly. The FEM calculation shows a similar electric field distribution compared to that of the P-CS-SETENG, which leads to similar V_{OC} and Q_{SC} profiles (Figure S22a-b) with only a little decrease in magnitude (Figure S26a-b). The V_{OC} increases in sequence when the reference electrode is located at below, below right, right and upper right of the primary electrode. The Q_{SC} for the reference electrode in different directions are also in the same rank, but the differences among them are relatively small. Furthermore, the average power under different load resistors of the D-CS-SETENG with reference electrode located at different directions is calculated (Figure S22c-d). The maximum average power and optimum load resistor of D-CS-SETENGs with reference electrodes located at below right, right and upper right are significantly higher than that at below (Figure S22e). Note that the V-Q plot corresponding to the maximum average power of D-CS-SETENGs can be found in Figure S22f. When the reference electrode is located at different locations, the V_{OCmax}, Q_{SCmax}, and maximum

average power of the D-CS-SETENG can be arrange from the largest to the smallest as follow: upper right, right, below right and below (Figure S26a-c).

Then, the influence of the reference electrode on the output performance of D-CS-SETENGs at *g* = 30 mm is also investigated through FEM simulation. It is found that on the whole, the output performance is improved compared with that at g = 1 mm (Figure S23 and Figure S26a-f). When the reference electrode is located at different locations, the V_{OCmax}, Q_{SCmax}, and maximum average power of the D-CS-SETENG can be arrange from the largest to the smallest as follow: upper, upper right, right, below right and below (Figure S26d-f). Similar to P-CS-SETENGs, under such a large gap, the significant performance gain is only obtained with the upper-located reference electrode, while the performance differences among all other directions are very small.

Analysis and optimization for D-S-SETENGs.

The D-S-SETENG has a similar electric field distribution to the P-S-SETENG, resulting in similar V_{OC}, Q_{SC}, average power and V-Q plot with only a little decrease in magnitude (Figure S24-S25, and Figure S26g-l). In the case of g=1 mm, the P-S-SETENG can obtain the maximum output performance (V_{OC}, Q_{SC} and average power) when the reference electrode is located at the right of the primary electrode, followed by the below right and upper right, and finally the other directions (Figure S24). The V_{OCmax}, Q_{SCmax} and maximum average power of the D-S-SETENG are shown in Figure S26g-i, which share the same changing trend but are smaller in magnitude compared with those of the P-S-SETENG. As for g=30 mm, the V_{OCmax}, Q_{SCmax} and maximum average power of the D-S-SETENG have little difference when the reference electrode is located at different directions (Figure S25), and all of them also share the same changing trend but are smaller in magnitude compared with those of the P-S-SETENG (Figure S26j-l).

In summary, the influences of the reference electrode location on the D-CS-SETENG and D-S-SETENG and are very similar to those on the P-CS-SETENG and P-S-SETENG, respectively. Hence, the optimization strategies for the reference electrode design proposed in Section 2.1 and 2.2 are also applicable to the D-CS-SETENG and D-S-SETENG.

Figure S1. (a) The electric field intensity distribution diagram in the P-CS-SETENG, where the size of the arrow indicates the strength of the electric field. (b-e) The distribution diagram of equipotential lines of the P-CS-SETENG when the reference electrode is located at the (b) below, (c) below right, (d) right, and (e) upper right of the primary electrode.

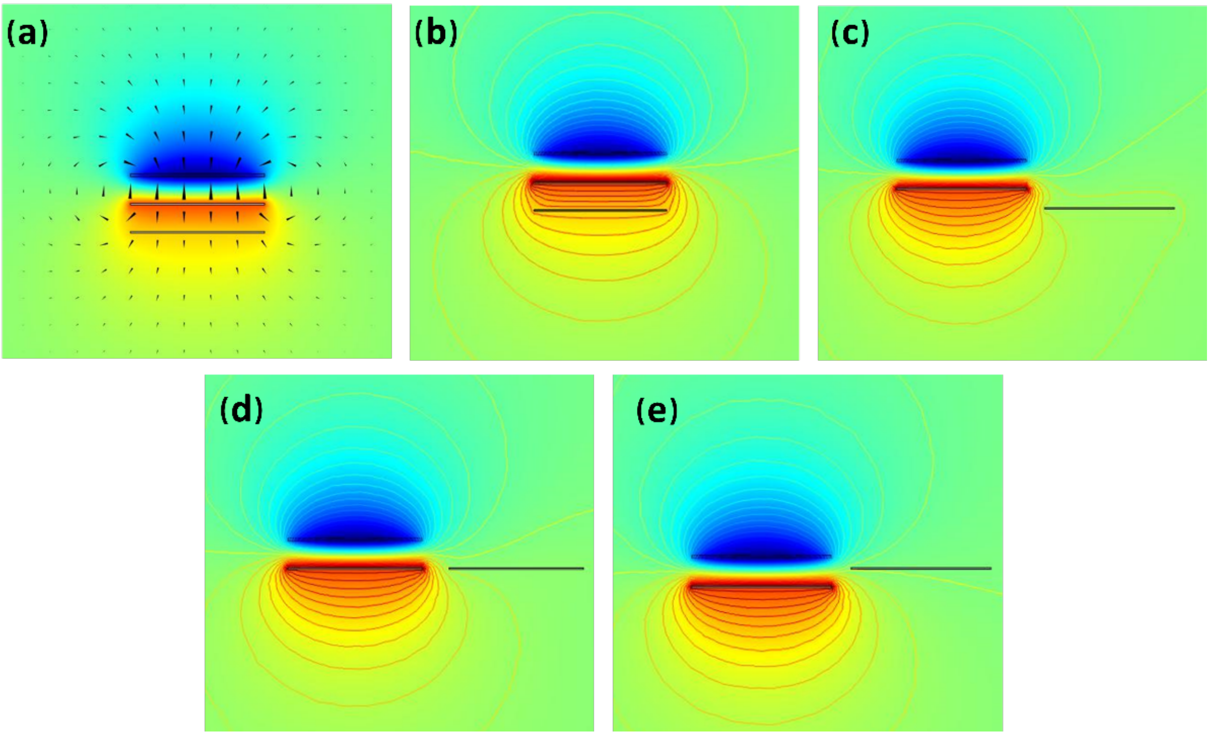

Figure S2. The V_{OC} and Q_{SC} of the P-CS-SETENGs at *g* of (a) 1 mm, (b) 2 mm, (c) 5 mm, (d) 10 mm, (e) 20 mm, and (f) 30 mm.

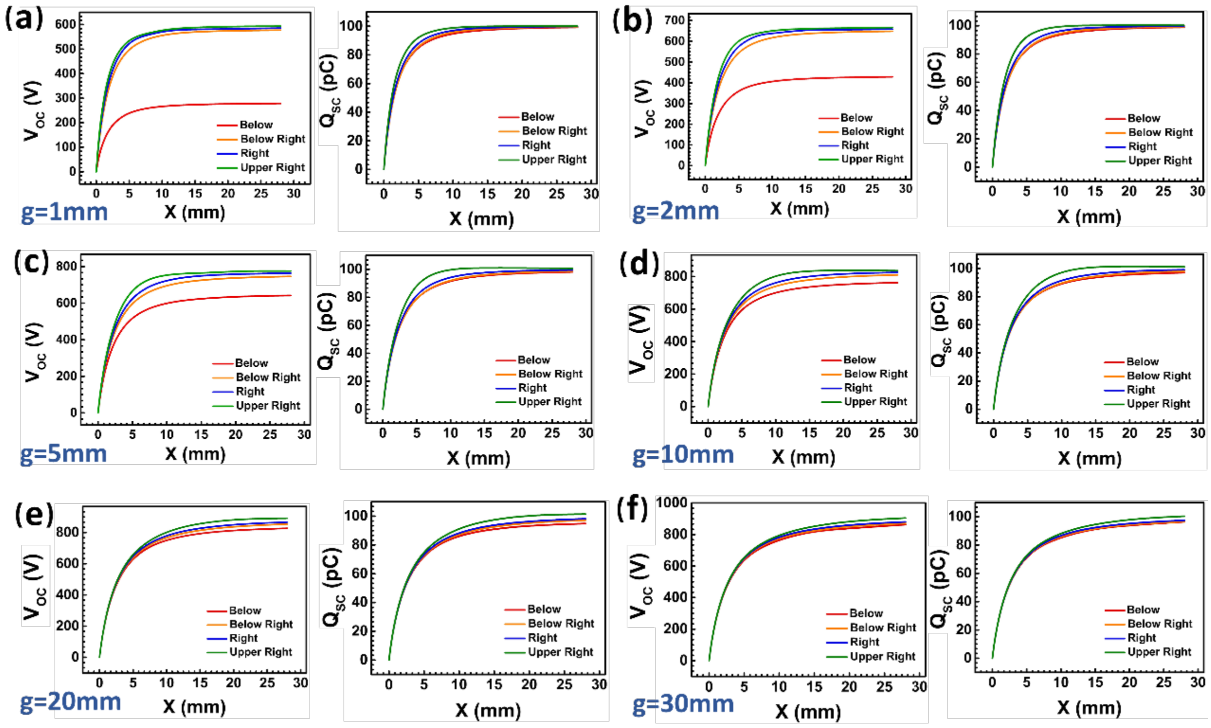

Figure S3. The (a) maximum voltage, (b) maximum current, and (c) average power of P-CS-SETENGs with the reference electrode located at different directions when g = 1 mm.

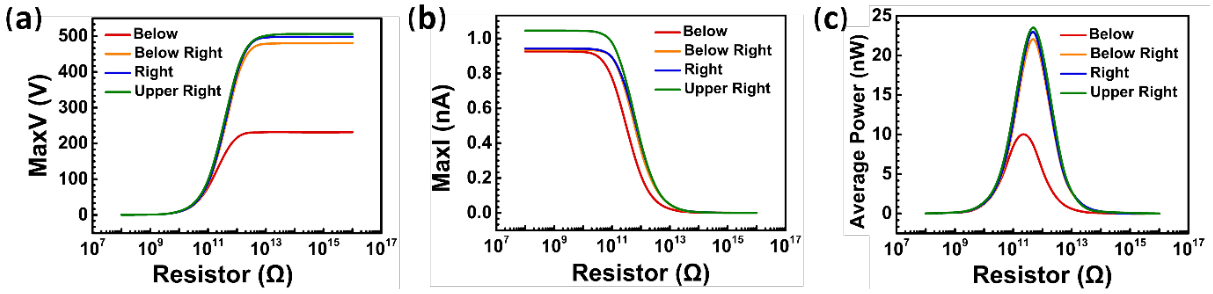

Figure S4. The (a) V_{OC}, (b) Q_{SC}, and (c) $V_{OC\ max}$ and $Q_{SC\ max}$ of P-CS-SETENGs with the reference electrode located at different directions when g = 30 mm.

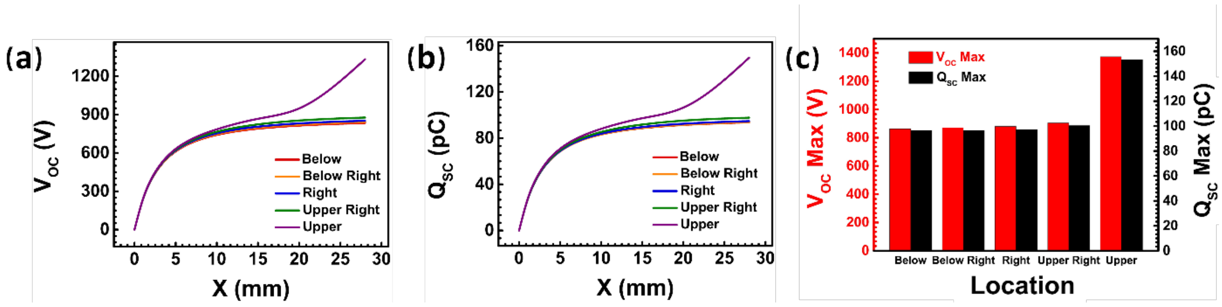

Figure S5. The (a) maximum voltage, (b) maximum current, and (c) average power of P-CS-SETENGs when the reference electrode is located at different directions (g = 30 mm).

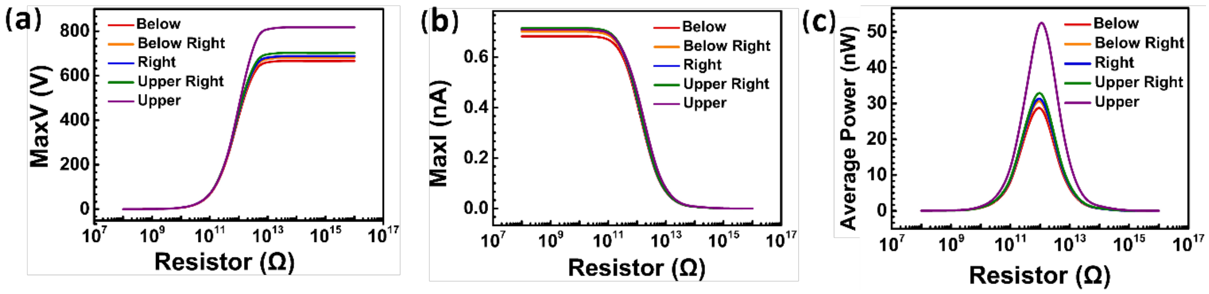

Figure S6. The (a) V_{OC}, (b) Q_{SC}, and (c) average power of P-CS-SETENGs when the reference electrode area is different (g = 1 mm).

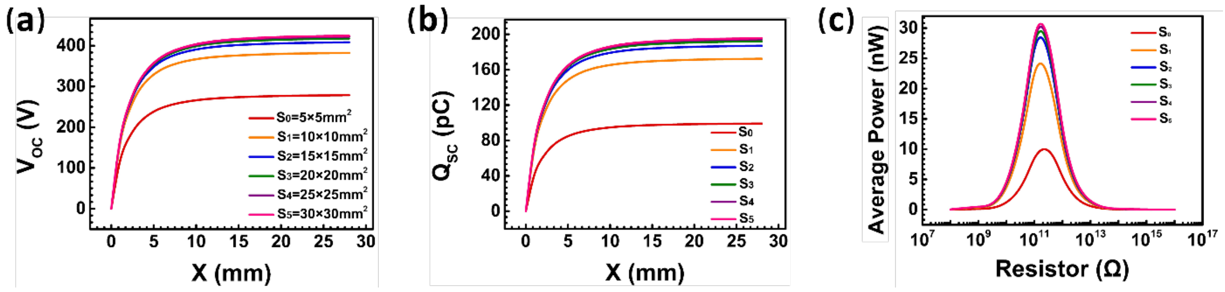

Figure S7. (a) The distribution diagram of equipotential lines in the electric field of the P-CS-SETENG when the reference electrode with an area of 5×5 mm^2 is located below the primary electrode (g = 1 mm). (b) The distribution diagram of equipotential lines of the P-CS-SETENG when the area of the reference electrode increases (g = 1 mm). (c) The distribution diagram of equipotential lines of the P-CS-SETENG when the thickness of the reference electrode increases (g = 1 mm).

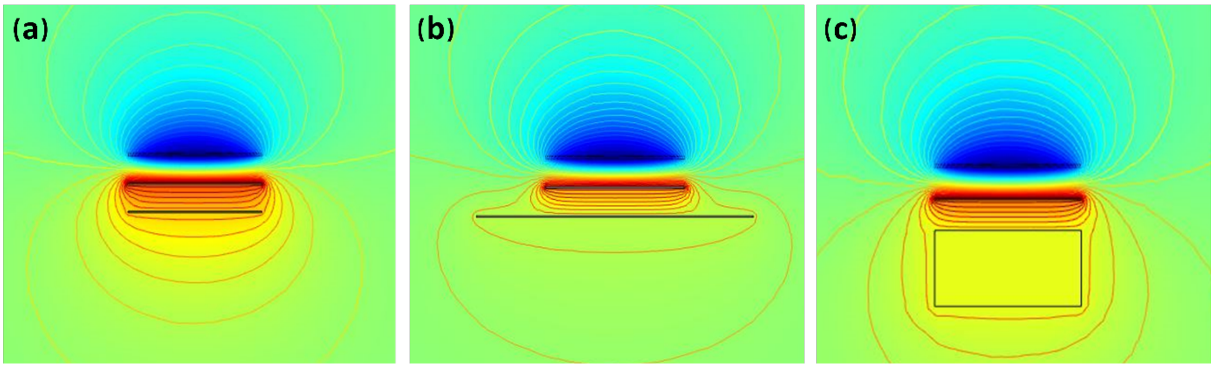

Figure S8. The equivalent electrical circuit model based on capacitor behavior.

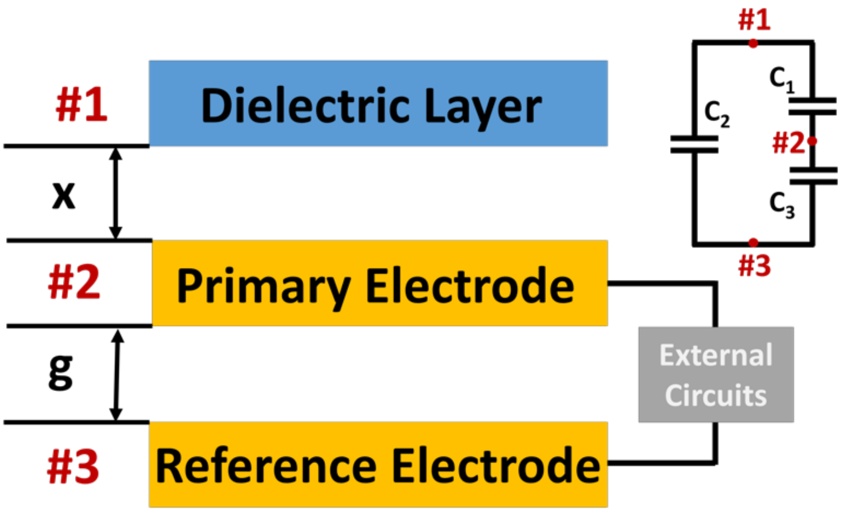

Figure S9. (a-c) The output performance of the P-CS-SETENG when the reference electrode area is different at g = 2 mm, including the (a) V_{OC}, (b) Q_{SC}, and (c) average power over a wider range of load resistor loads. (d-f) The output performance of the P-CS-SETENG when the reference electrode area is different at g = 5 mm. (g-i) The output performance of the P-CS-SETENG when the reference electrode area is different at g = 10 mm. (j-l) The output performance of the P-CS-SETENG when the reference electrode area is different at g = 15 mm.

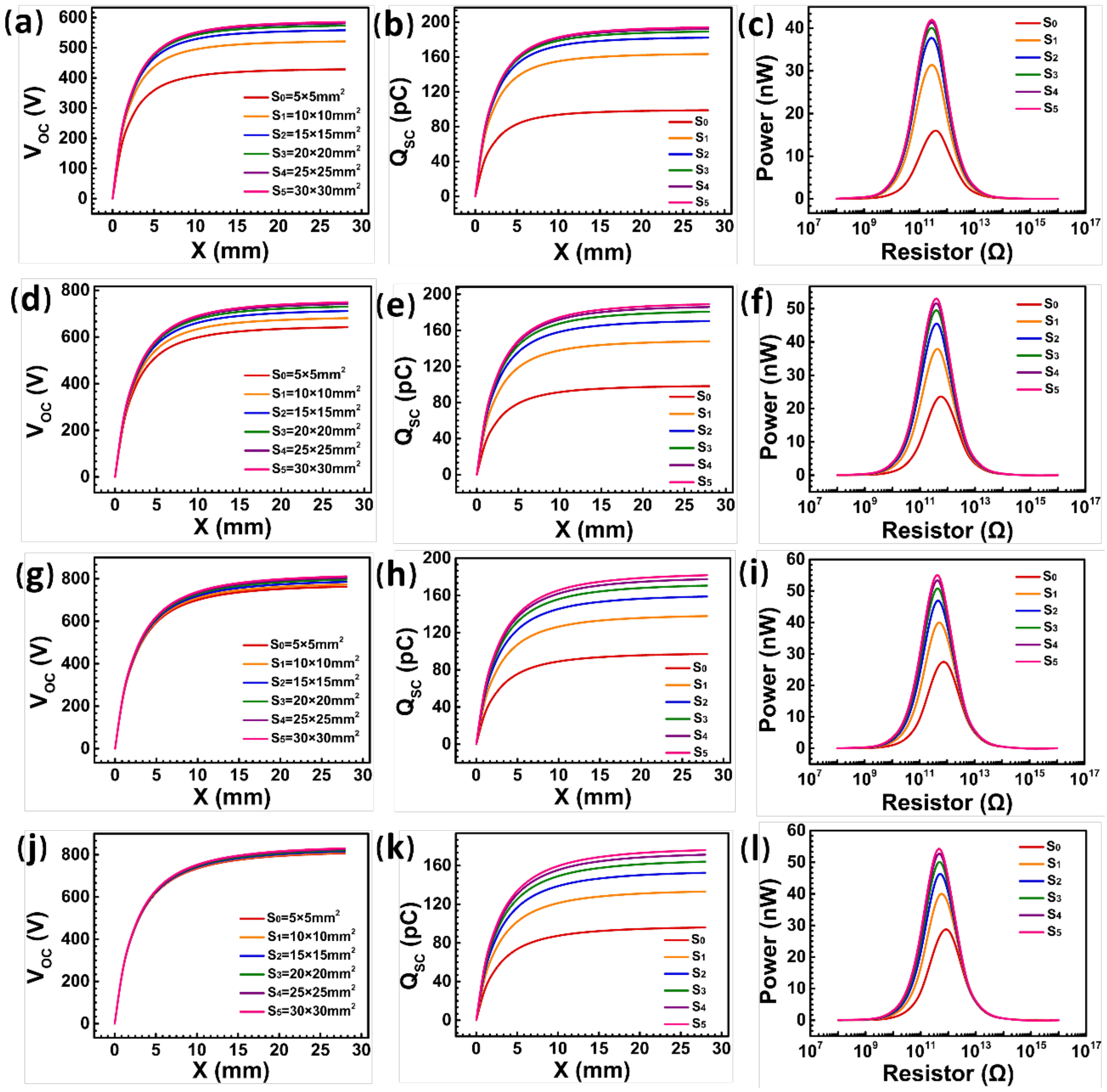

Figure S10. The (a) V_{OC}, (b) Q_{SC}, and (c) average power of the P-CS-SETENG when the reference electrode thickness is different (g = 1 mm).

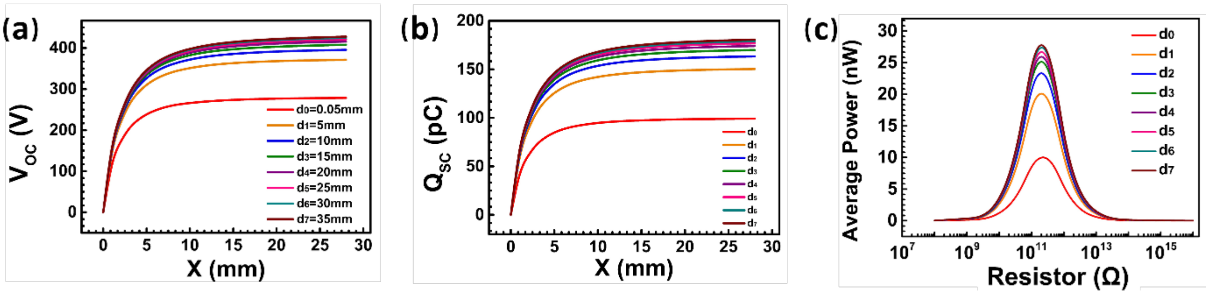

Figure S11. The (a) V_{OC}, (b) Q_{SC}, and (c) V-Q closed loop under optimum load resistor of P-CS-SETENGs under various Models.

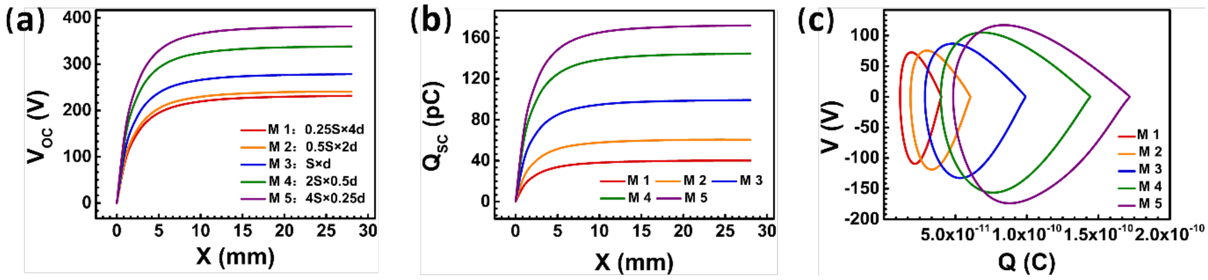

Figure S12. (a-c) The output performance of the P-CS-SETENG with a reference electrode volume of 4 V and different α (α=S/d^2), including the (a) V_{OC}, (b) Q_{SC} and (c) average power over a wider range of load resistors (g = 1 mm). (d-f) The output performance of the P-CS-SETENG with a reference electrode volume of 9 V and different α, including the (d) V_{OC}, (e) Q_{SC}, and (f) average power over a wider range of load resistors (g = 1 mm).

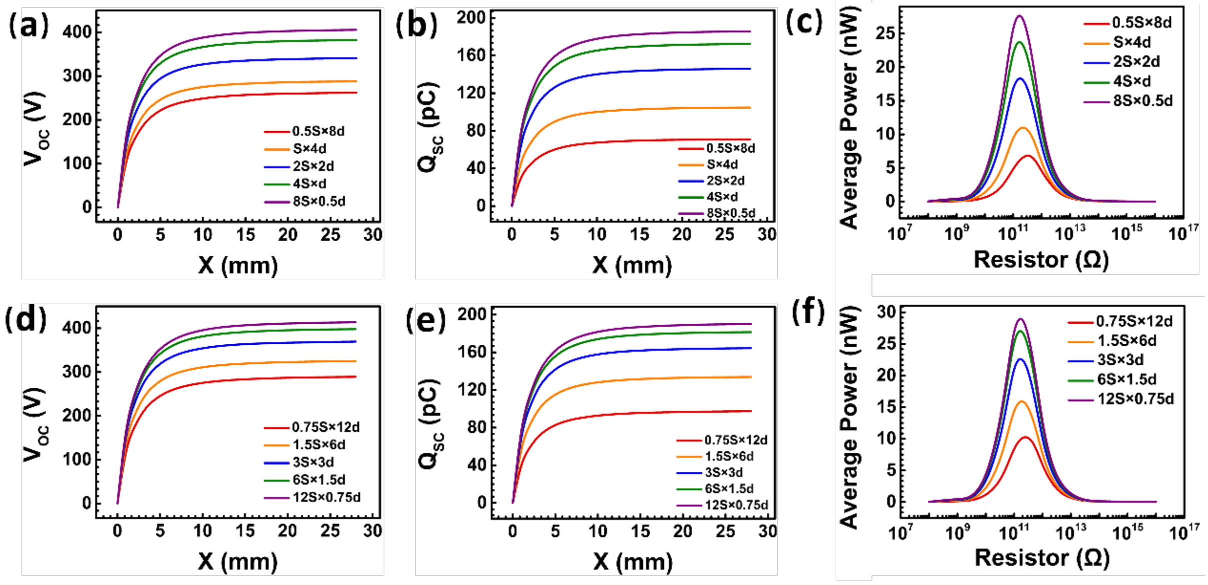

Figure S13. The schematic diagram of the reference electrodes in different shapes, including (a) triangular pyramid, (b) rectangular pyramid, (c) cone, (d) triangular prism, (e) quadrangular, (f) cylinder, and (g) sphere.

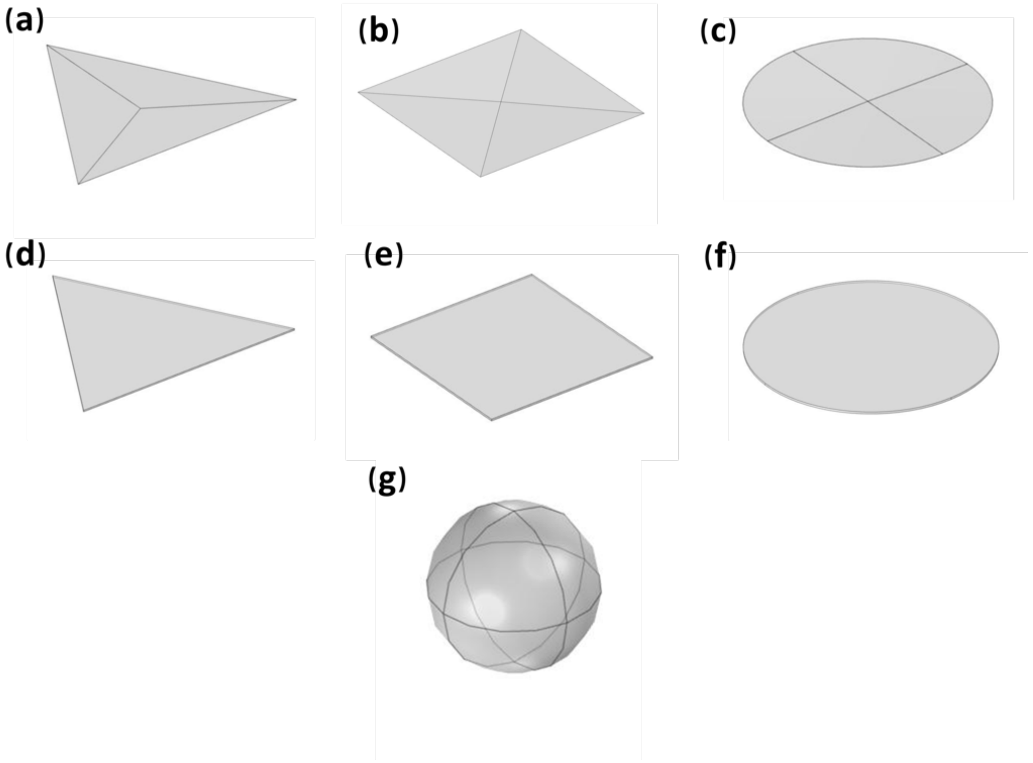

Figure S14. The (a) V_{OC}, (b) Q_{SC}, (c) $V_{OC\ max}$ and $Q_{SC\ max}$, (d) average power, (e) maximum average power and optimum load resistor, and (f) V-Q closed loop under optimum load resistor with reference electrodes having the same volume and the same thickness in different shapes.

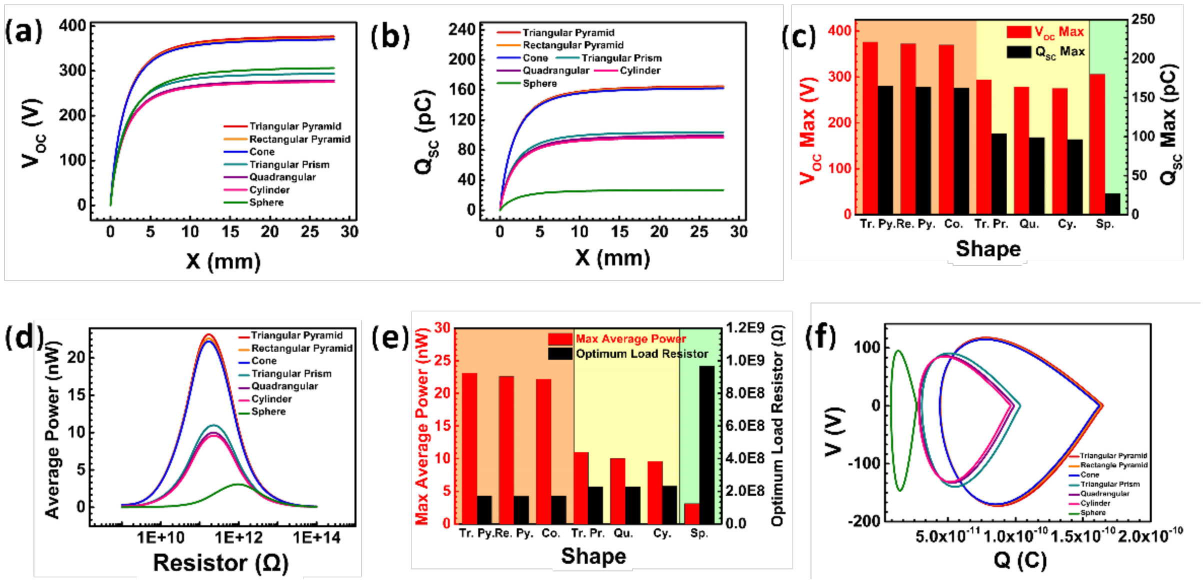

Figure S15. The (a) V_{OC}, (b) Q_{SC}, (c) $V_{OC\ max}$ and $Q_{SC\ max}$, (d) average power, (e) maximum average power and optimum load resistor, and (f) V-Q closed loop under optimum load resistor with reference electrodes having the same volume and the same area in different shapes.

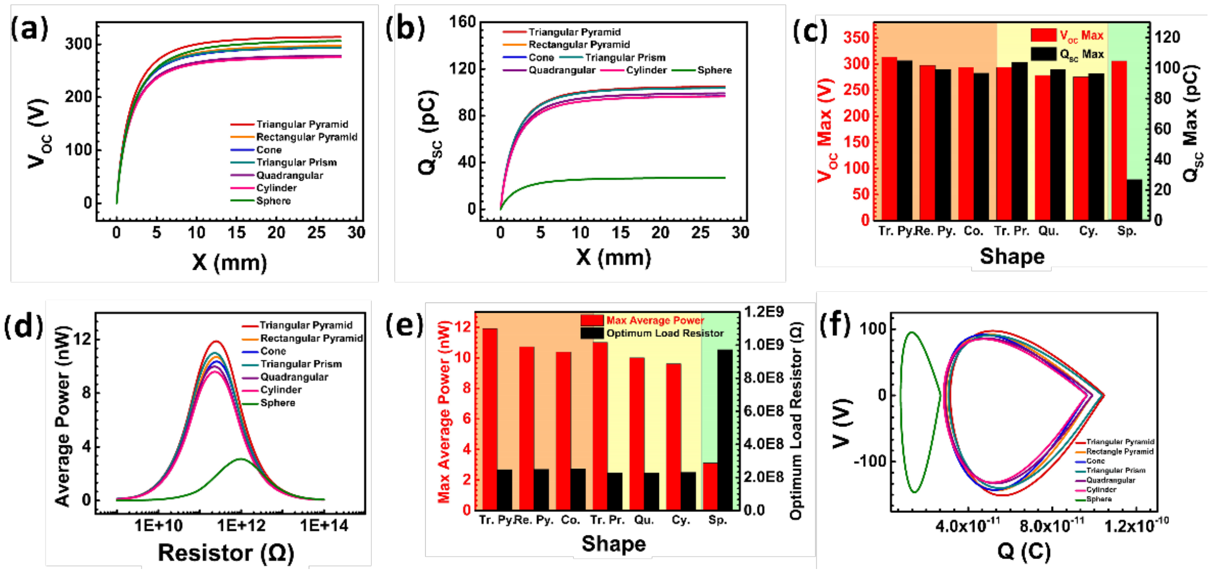

Figure S16. The (a) V_{OC}, (b) Q_{SC}, (c) average power, (d) average power over a wider range of load resistors, and (e) V-Q closed loop under optimum load resistor of P-S-SETENGs when the reference electrode is located at different directions at g = 1 mm.

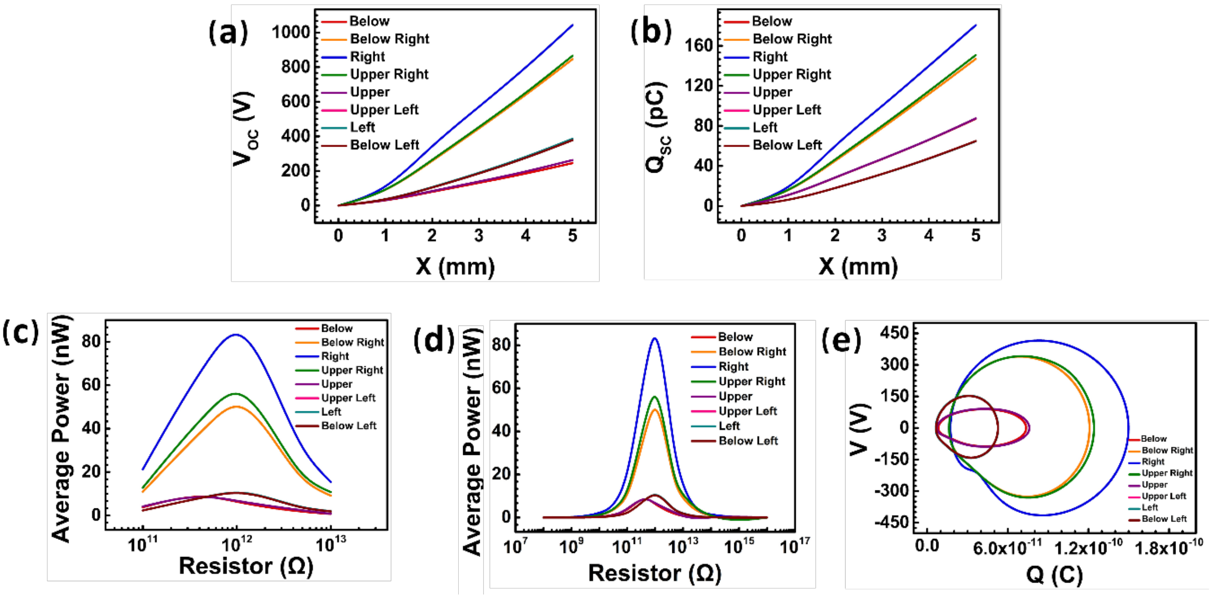

Figure S17. (a) The structural diagram of the P-S-SETENG. (b) The distribution diagram of equipotential lines in the electric field of the P-S-SETENG.

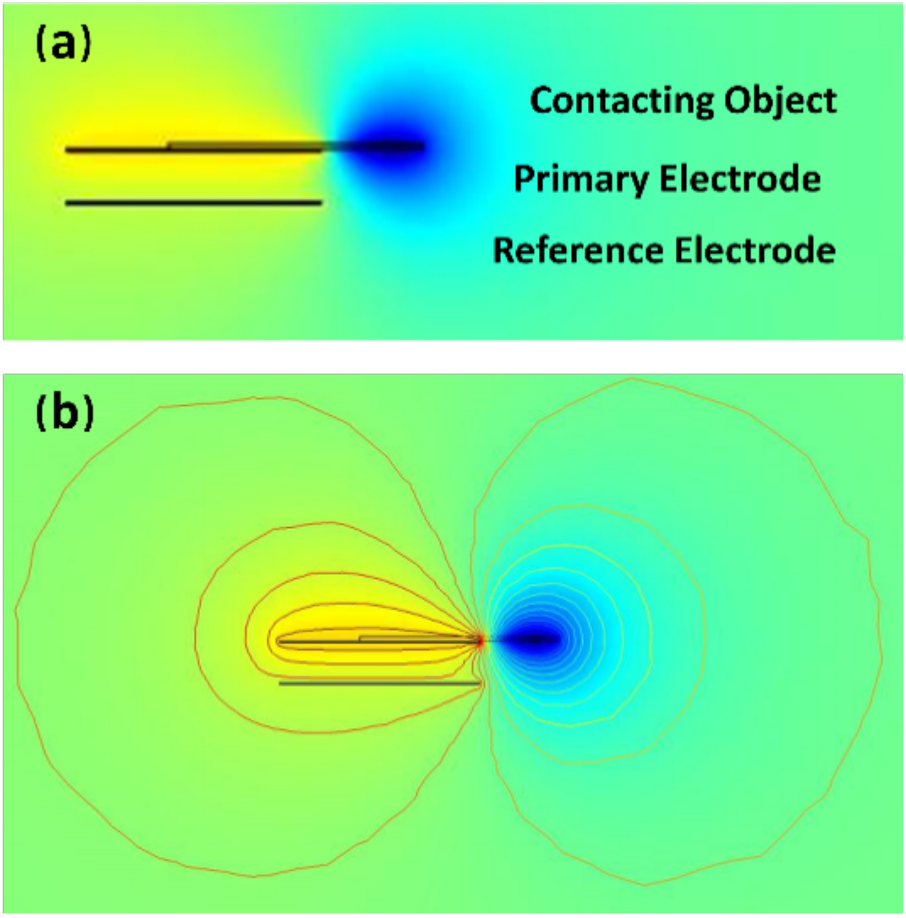

Figure S18. The (a) V_{OC}, (b) Q_{SC}, (c) average power, (d) average power over a wider range of load resistors, and (e) V-Q closed loop under optimum load resistor of P-S-SETENGs when the reference electrode is located at different directions at g = 30 mm.

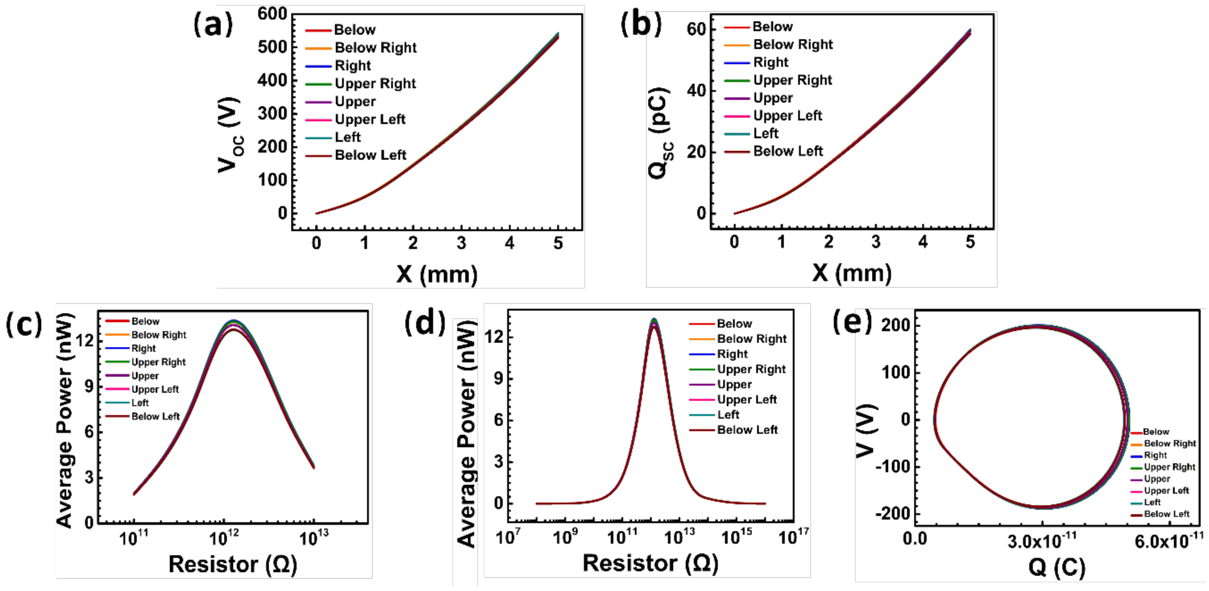

Figure S19. The (a) V_{OC}, (b) Q_{SC}, (c) average power, (d) average power over a wider range of load resistors, and (e) V-Q closed loop under optimum load resistor of P-S-SETENGs when the area of the reference electrode is different (g = 1 mm).

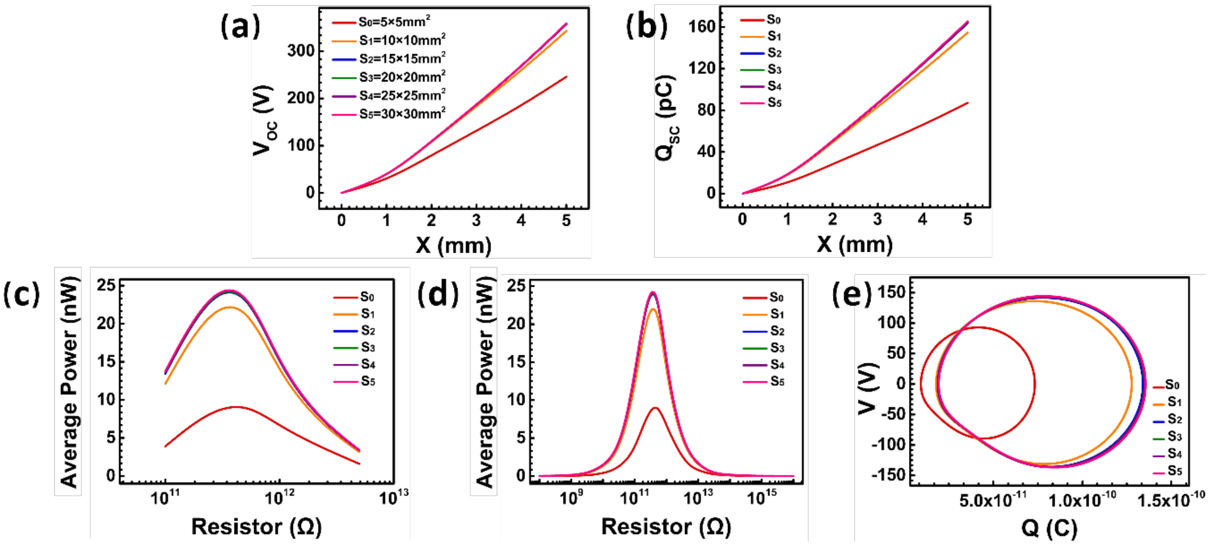

Figure S20. The (a) V_{OC}, (b) Q_{SC}, (c) average power, (d) average power over a wider range of load resistors, and (e) V-Q closed loop under optimum load resistor of P-S-SETENGs when the thickness of the reference electrode is different (g = 1 mm).

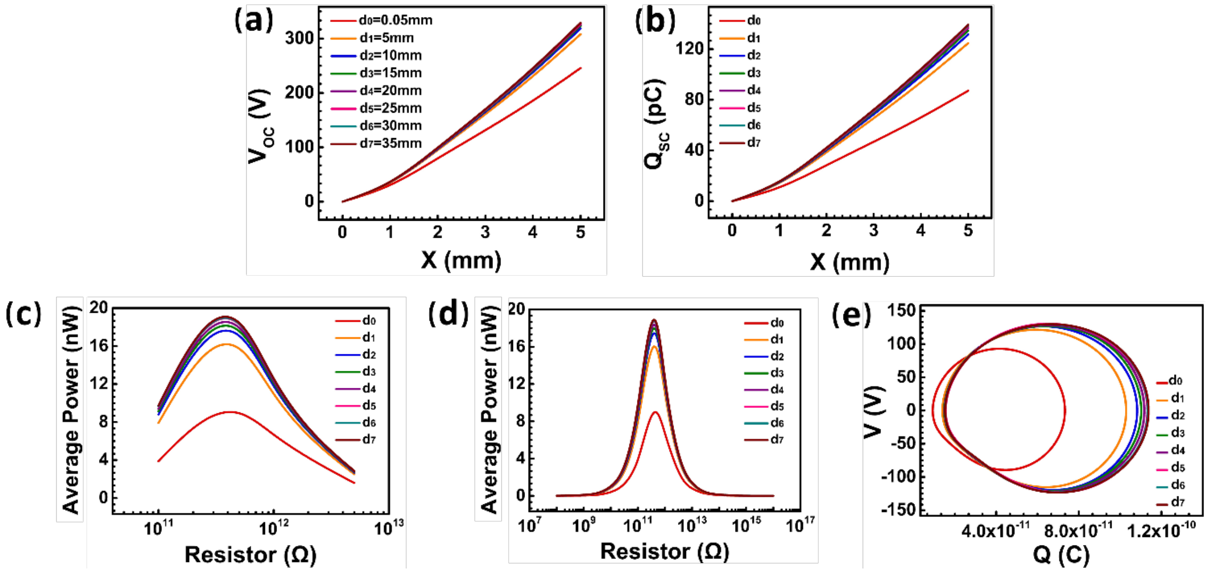

Figure S21. The structural diagram of the (a) D-CS-SETENG and (b) D-S-SETENG.

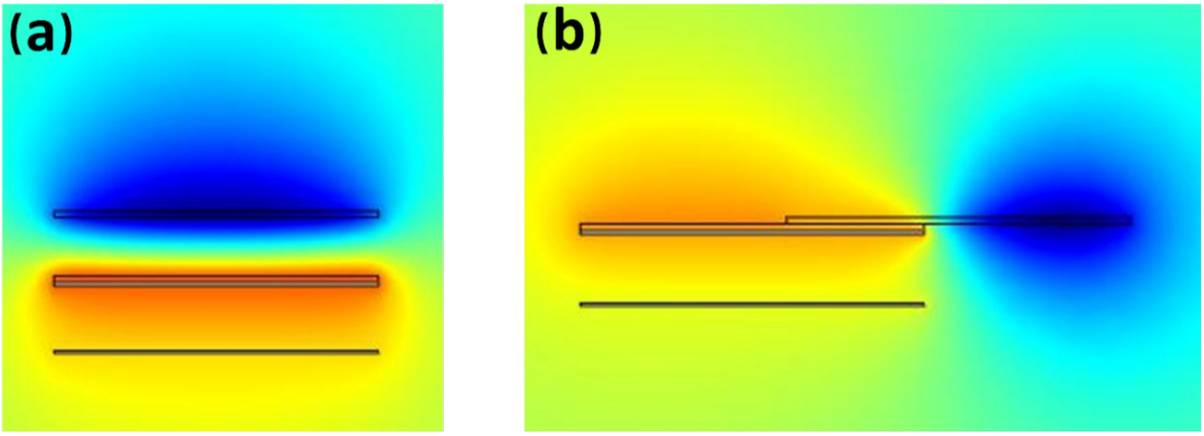

Figure S22. The (a) V_{OC}, (b) Q_{SC}, (c) average power, (d) average power over a wider range of load resistors, (e) maximum average power and optimum load resistor, and (f) V-Q closed loop under optimum load resistor of D-CS-SETENGs when the reference electrode is located at different directions at g = 1 mm.

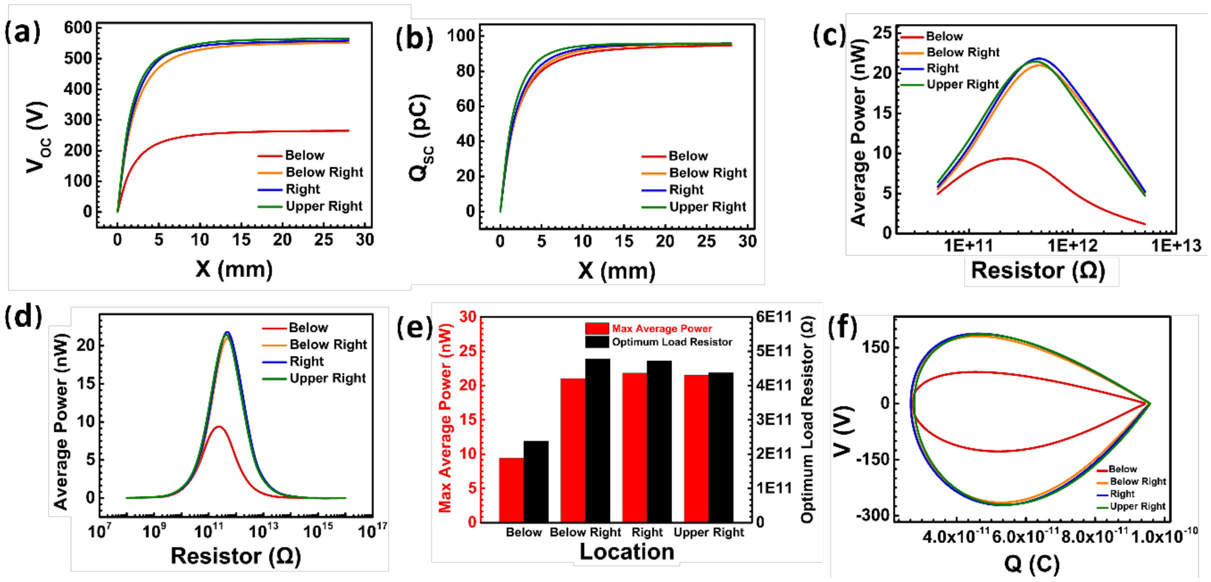

Figure S23. The (a) V_{OC}, (b) Q_{SC}, (c) average power, (d) average power over a wider range of load resistors, (e) maximum average power and optimum load resistor, and (f) V-Q closed loop under optimum load resistor calculated by D-CS-SETENGs when the reference electrode is located at different directions at g = 30 mm.

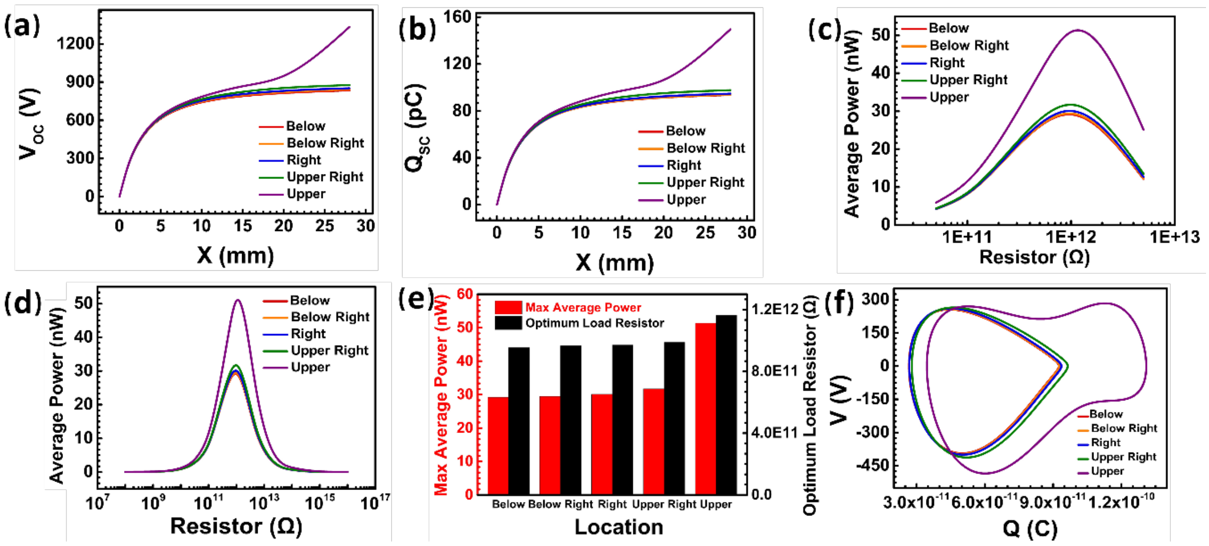

Figure S24. The (a) V_{OC}, (b) Q_{SC}, (c) average power, (d) average power over a wider range of load resistors, and (e) V-Q closed loop under optimum load resistor of D-S-SETENGs when the reference electrode is located at different directions at g = 1 mm.

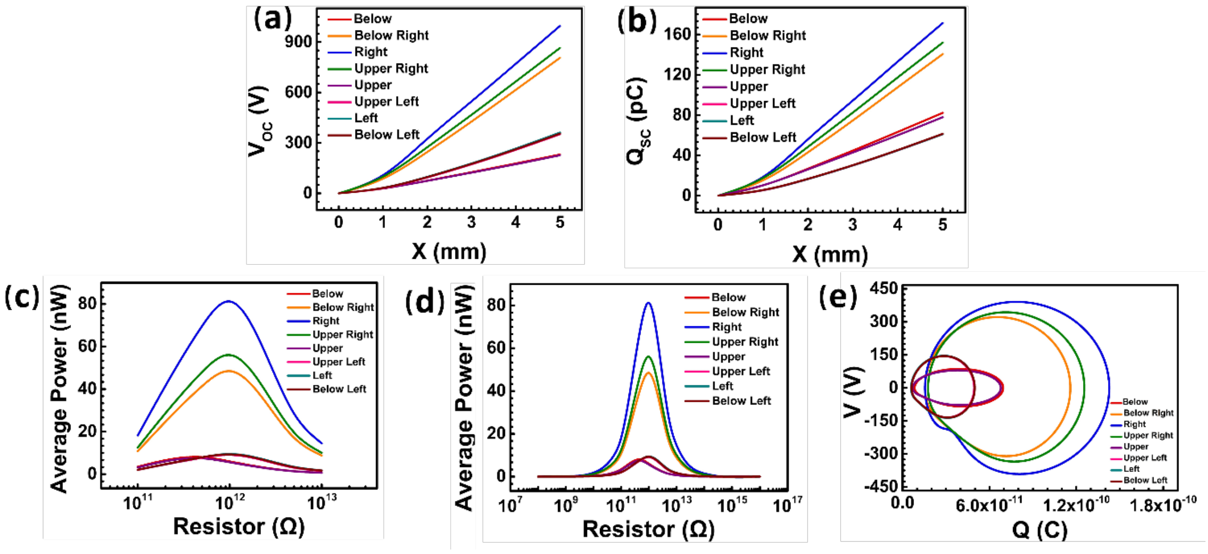

Figure S25. The (a) V_{OC}, (b) Q_{SC}, (c) average power, (d) average power over a wider range of load resistors, and (e) V-Q closed loop under optimum load resistor of D-S-SETENGs when the reference electrode is located at different directions at g = 30 mm.

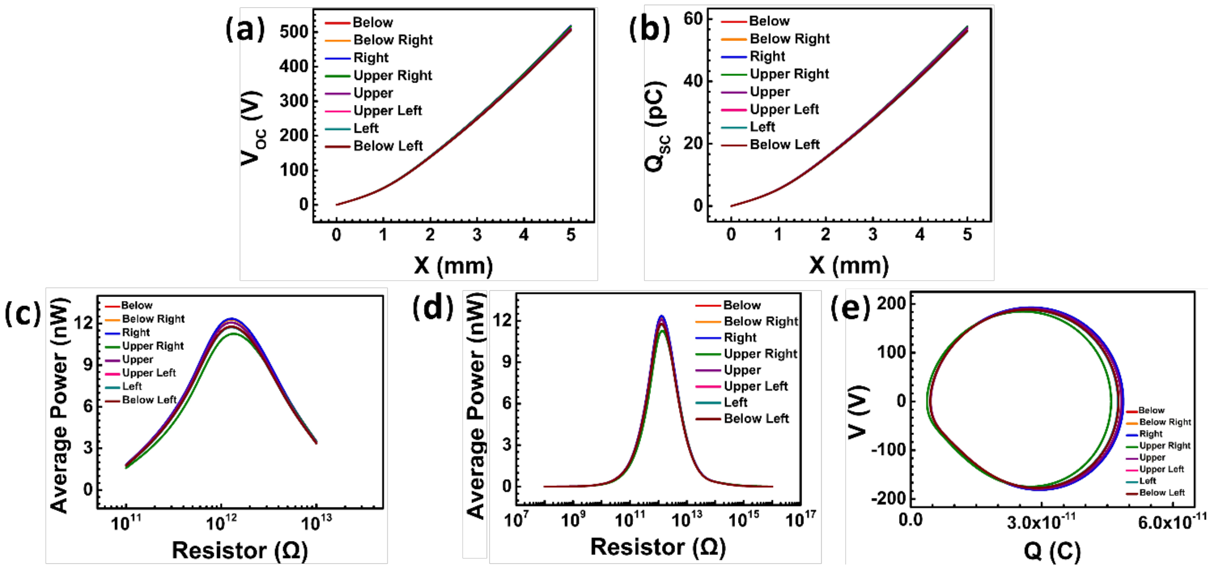

Figure S26. The effect of the reference electrode's location on the output performance of the D-CS-SETENG and D-S-SETENG. (a-c) The comparison of (a) V_{OCmax}, (b) Q_{SCmax}, and (c) maximum average power of P-CS-SETENGs and D-CS-SETENGs when the reference electrode is located in different directions at g = 1 mm. (d-f) The comparison of (d) V_{OCmax}, (e) Q_{SCmax}, and (f) maximum average power of P-CS-SETENGs and D-CS-SETENGs when the reference electrode is located in different directions at g = 30 mm. (g-i) The comparison of (g) V_{OCmax}, (h) Q_{SCmax}, and (i) maximum average power of P-S-SETENGs and D-S-SETENGs when the reference electrode is located in different directions at g = 1 mm. (j-l) The comparison of (j) V_{OCmax}, (k) Q_{SCmax}, and (l) maximum average power of P-S-SETENGs and D-S-SETENGs when the reference electrode is located in different directions at g = 30 mm.

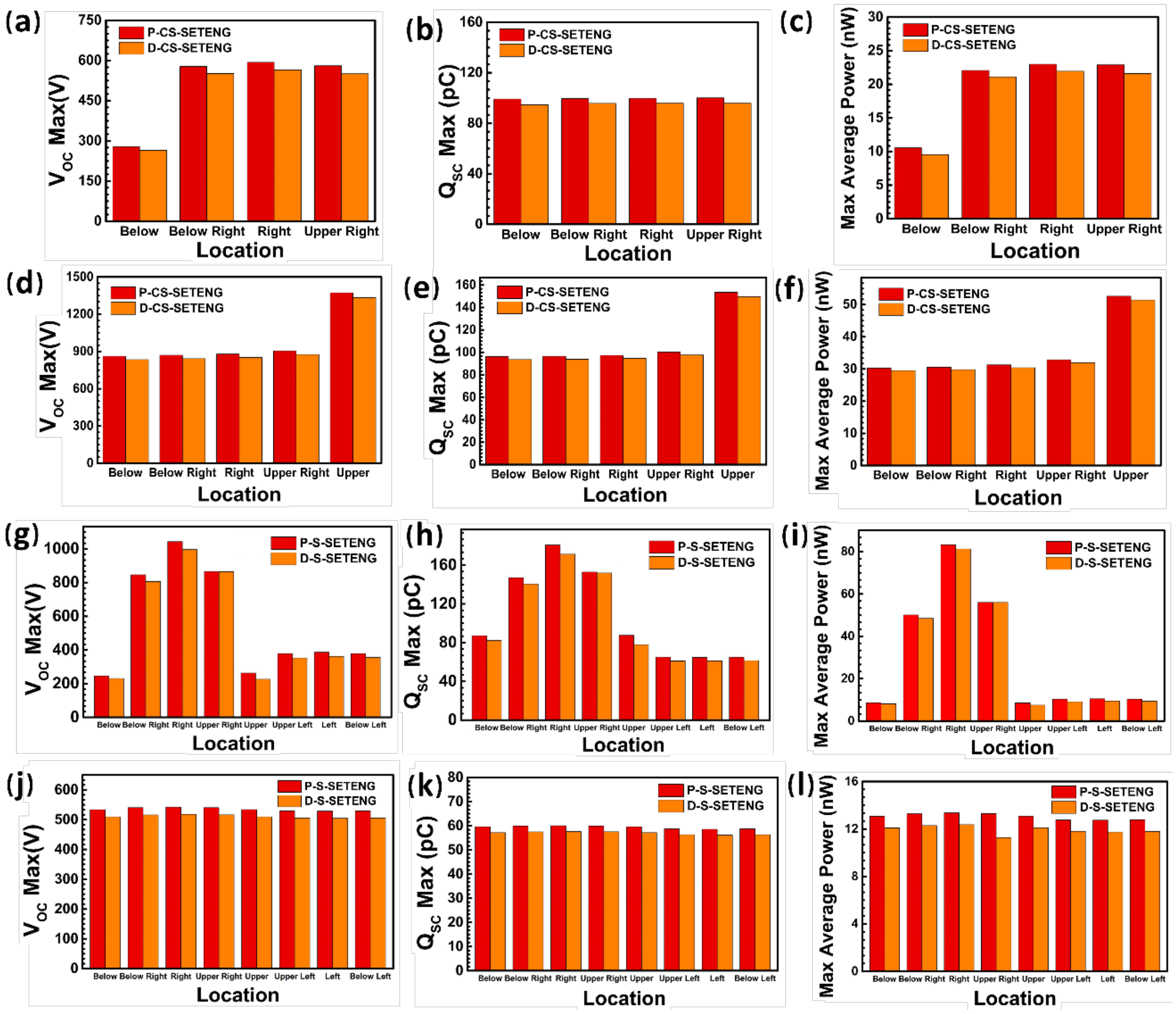

Figure S27. Images showing the reference electrode bending at different angles. (g=1 mm)

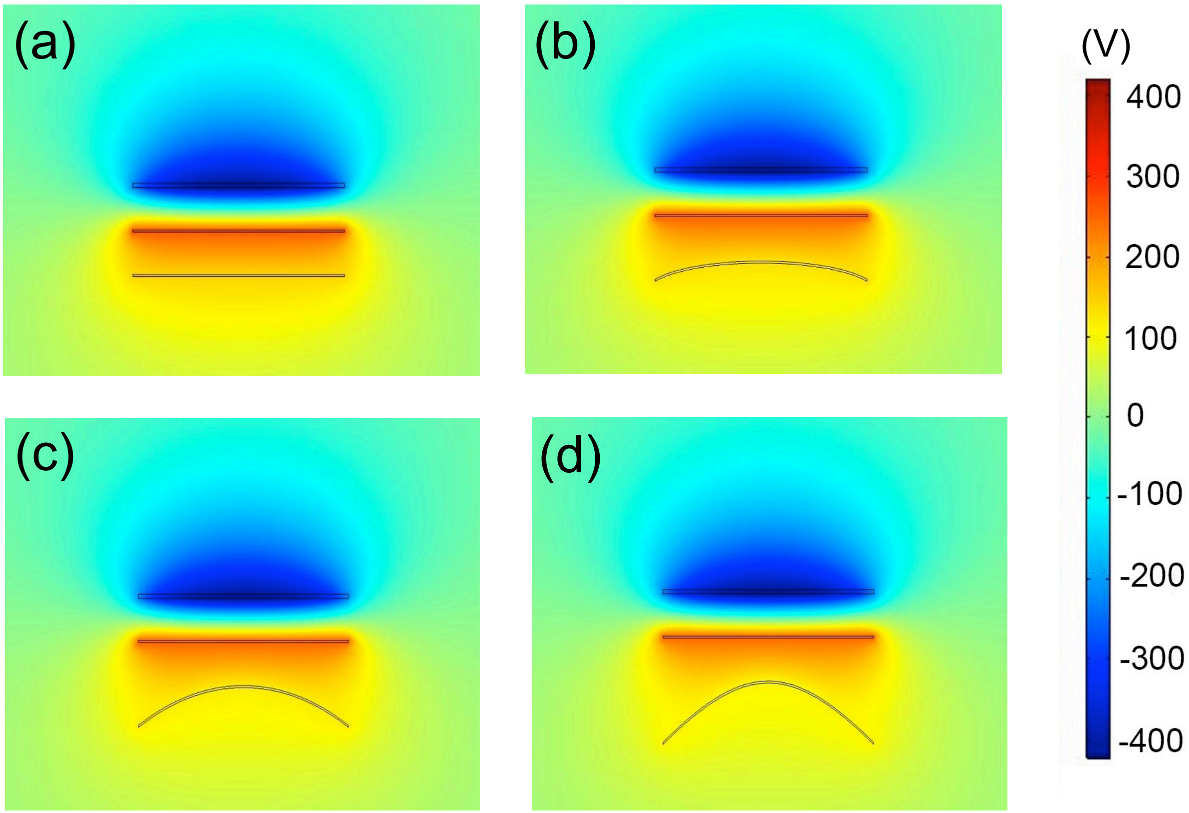

Figure S28. (a-c) The (a) open-circuit voltage (V_{OC}) and (c) short-circuit transferred charge (Q_{SC}) of the SETENG with the reference electrode bending at different angles. (b-d) The (b) V_{OCmax} and (d) Q_{SCmax} of the SETENG with the reference electrode bending at different angles. Note: g=1 mm, contact/separation mode.

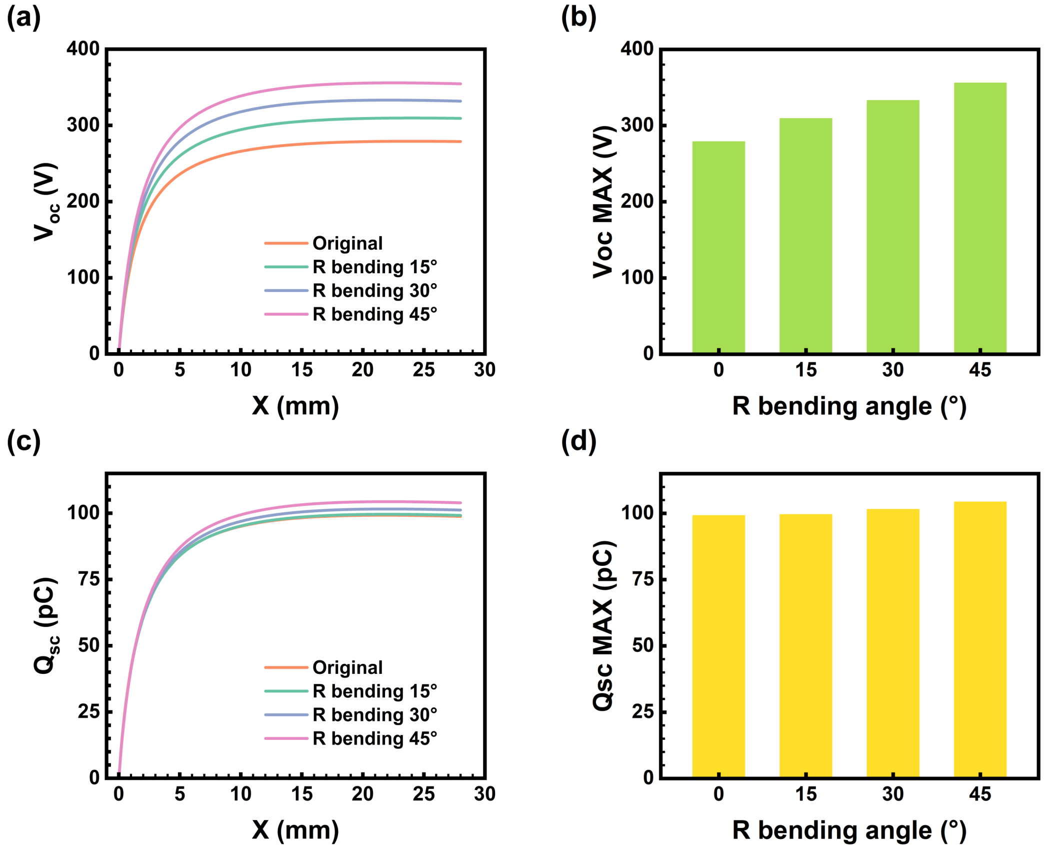

Figure S29. (a) The average power of the STENG with the reference electrode bending at different angles. (b) The maximum average power and optimum load resistor of the SETENG with the reference electrode bending at different angles. Note: g=1 mm, contact/separation mode.

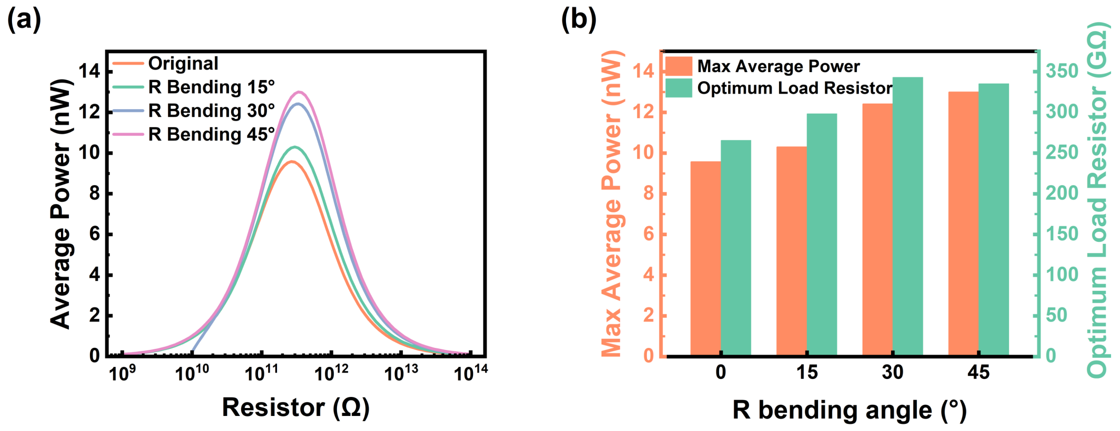

Figure S30. Images showing both the reference electrode and working part bending at different angles.

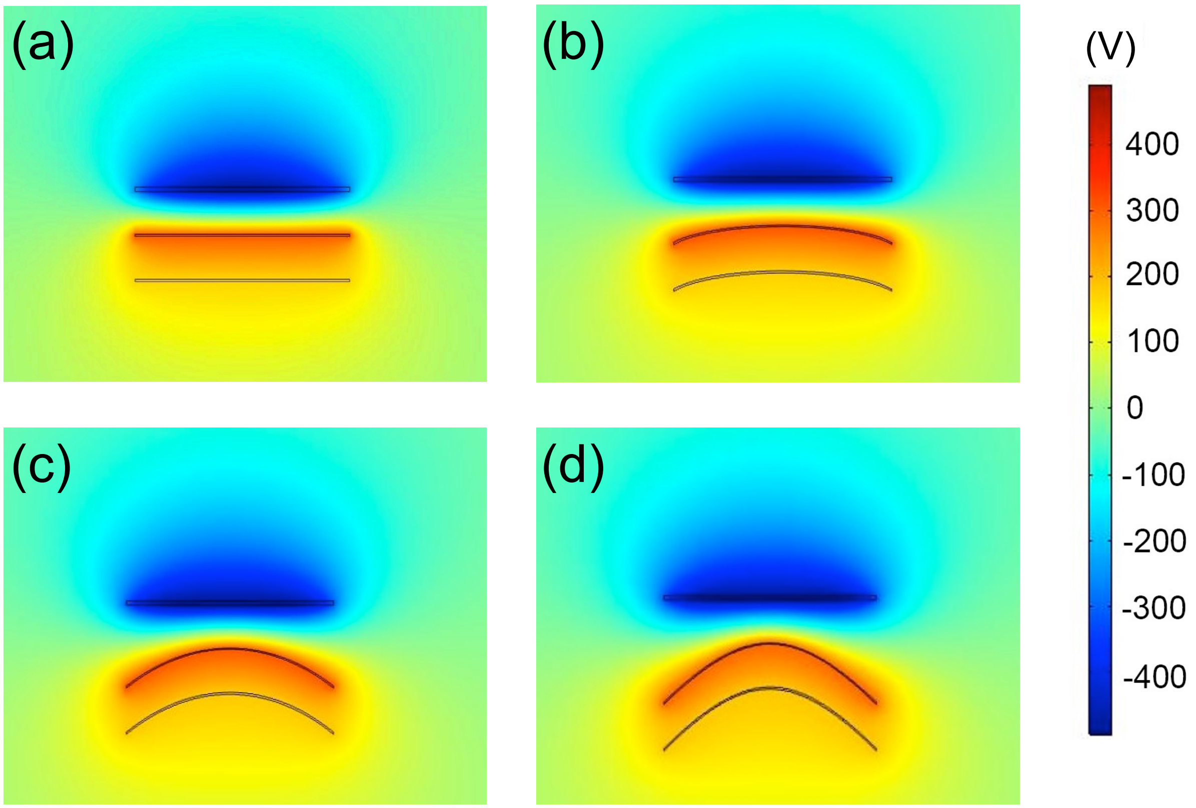

Figure S31. (a-c) The (a) open-circuit voltage (V_{OC}) and (c) short-circuit transferred charge (Q_{SC}) of the SETENG with both the reference electrode and working part bending at different angles. (b-d) The (b) V_{OCmax} and (d) Q_{SCmax} of the SETENG with both the reference electrode and working part bending at different angles. Note: g=1 mm, contact/separation mode.

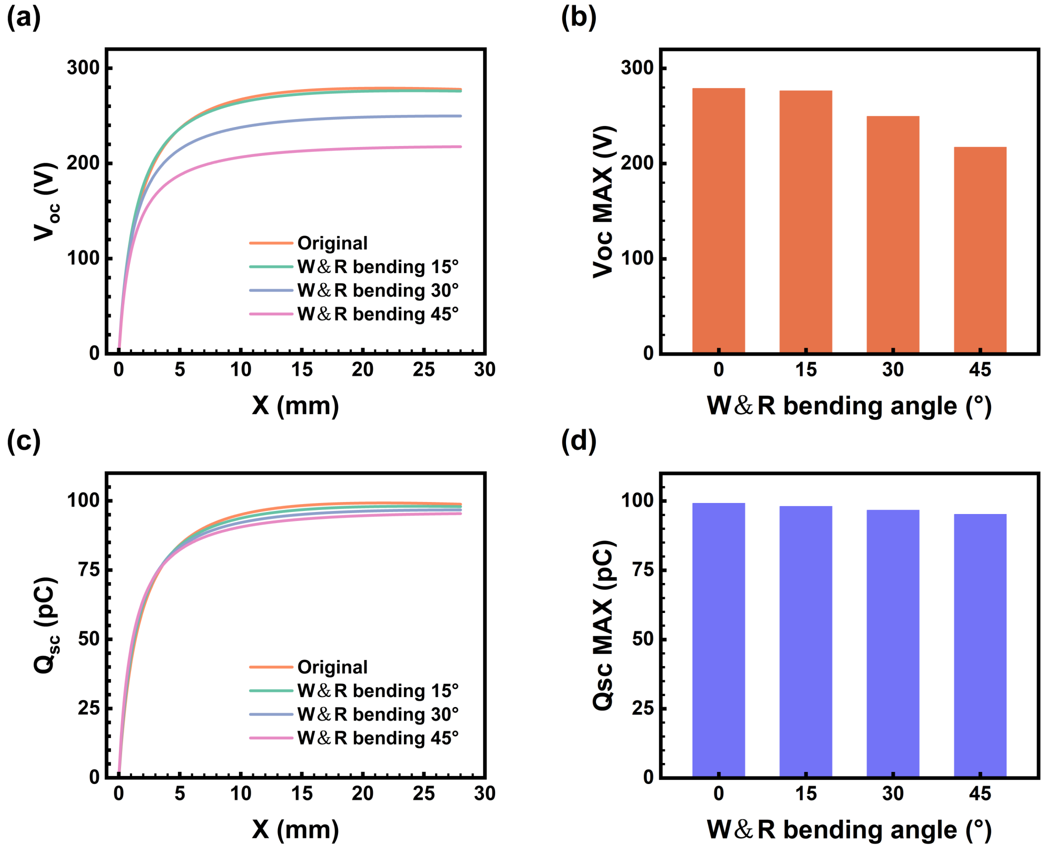

Figure S32. (a) The average power of the STENG with both the reference electrode and working part bending at different angles. (b) The maximum average power and optimum load resistor of the SETENG with both the reference electrode and working part bending at different angles. Note: g=1 mm, contact/separation mode.

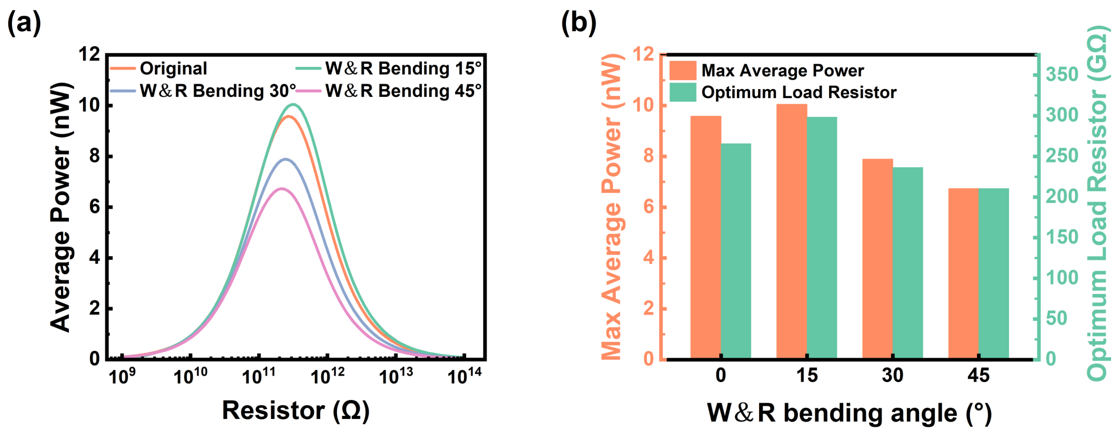

Figure S33. Experimental setup for the P-CS-SETENG and P-S-SETENG. (a) Images of the P-CS-SETENG. (b) Images of the P-S-SETENG.

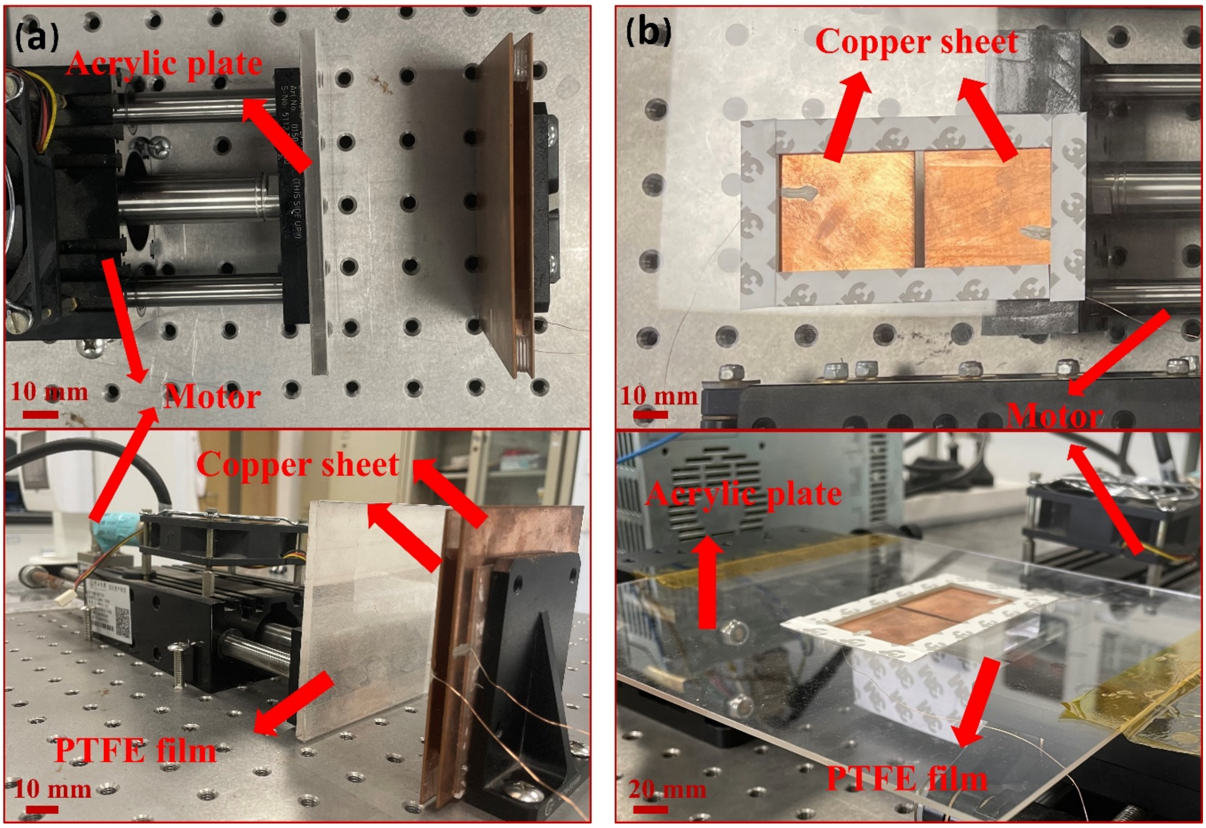

Figure S34. Brief schematic illustrations for the main optimization strategies.

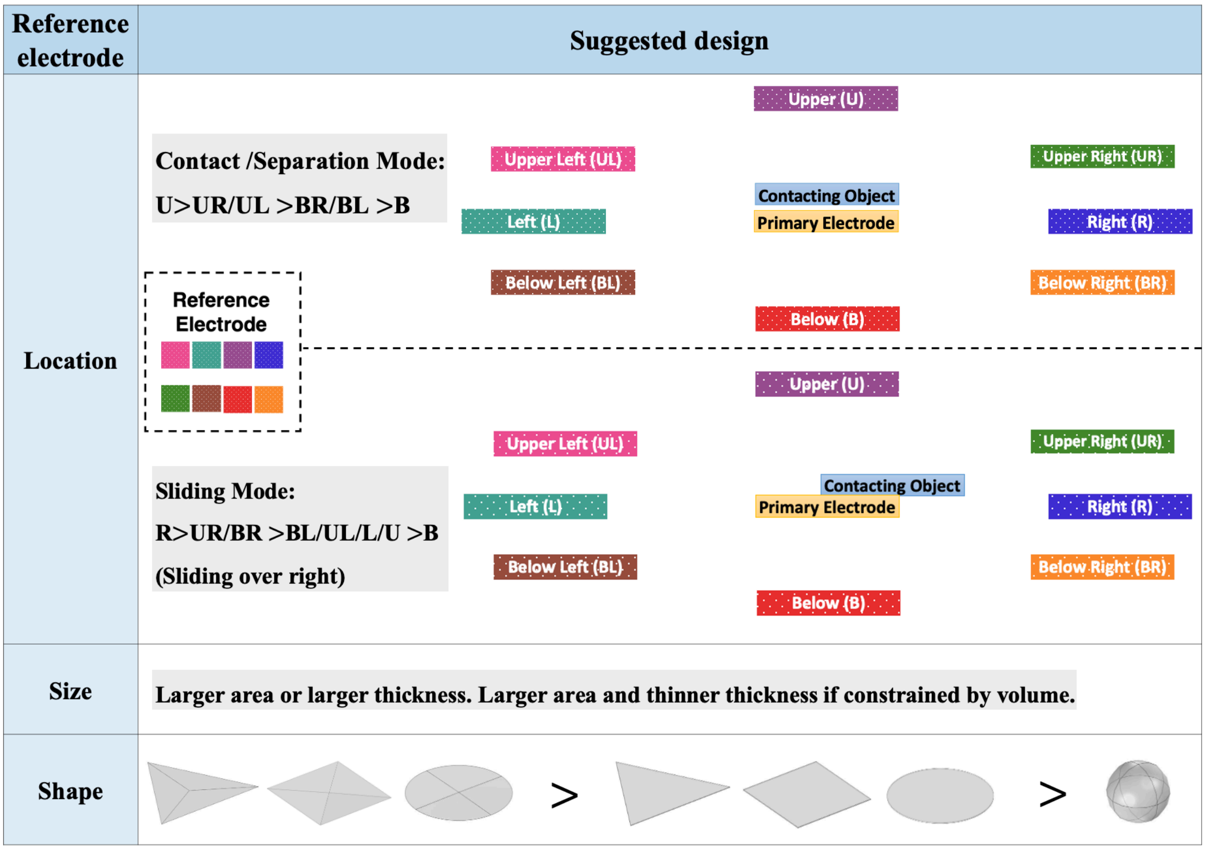

Note: The influences of the reference electrode's location, size and shape on the SETENG's performance decline with the increase in the gap between the primary and reference electrodes. In practice, if the reference electrode has to be placed directly under the primary electrode, to achieve high electric outputs, it is suggested that: (1) the gap between the primary and reference electrodes is preferred to be bigger; (2) the area and thickness are preferred to be larger; (3) the shape is preferred to be with sharp tips towards the primary electrode. In addition, since the electrical outputs gradually saturate with the increasing reference electrode size, excessive increase in the reference electrode size could be unnecessary especially when considering the fabrication cost. More detailed analysis and summarization of the optimization strategies for SETENGs are presented in the main text.

Figure S35. (a) The schematic diagram of the square plate capacitor with upper and lower plates having different lengths. (b) The mathematically simplified diagram of the derivation of capacitance formula.

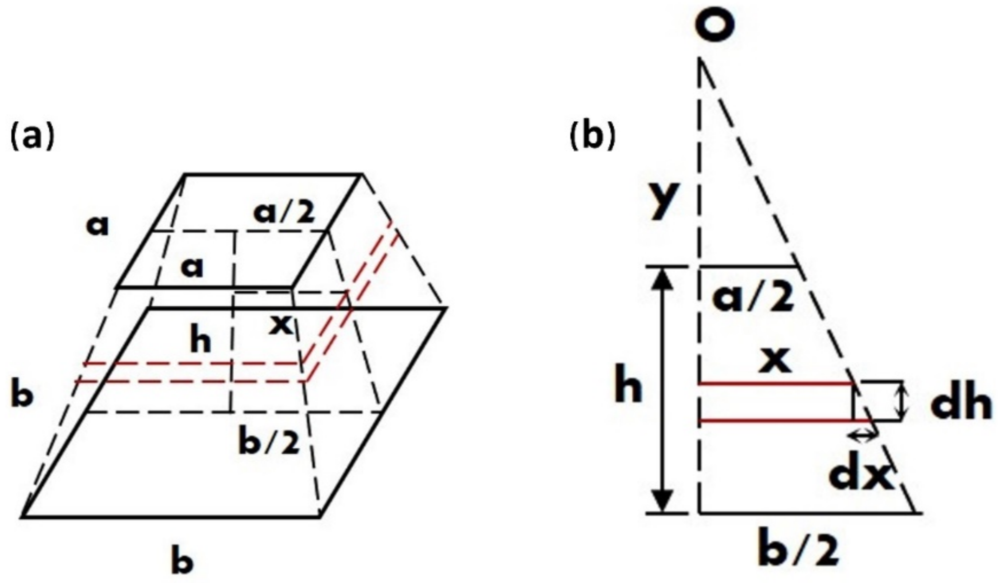

Supplement: Supplementary file 1 — Supporting Information [file ADVS-10-2206950-s001.pdf]
